# Adherence to the World Cancer Research Fund/American Institute for Cancer Research and Korean Cancer Prevention Guidelines and cancer risk: a prospective cohort study from the Health Examinees-Gem study

**DOI:** 10.4178/epih.e2023070

**Published:** 2023-08-01

**Authors:** Jeeyoo Lee, Aesun Shin, Woo-Kyoung Shin, Ji-Yeob Choi, Daehee Kang, Jong-Koo Lee

**Affiliations:** 1 Department of Preventive Medicine, Seoul National University College of Medicine, Seoul, Korea; 2 Cancer Research Institute, Seoul National University, Seoul, Korea; 3 Interdisciplinary Program in Cancer Biology Major, Seoul National University College of Medicine, Seoul, Korea; 4 Integrated Major in Innovative Medical Science, Seoul National University Graduate School, Seoul, Korea; 5 Medical Research Center, Genomic Medicine Institute, Seoul National University College of Medicine, Seoul, Korea; 6 Department of Biomedical Science, Seoul National University Graduate School, Seoul, Korea; 7 Institute of Health Policy and Management, Seoul National University Medical Research Center, Seoul, Korea; 8 JW LEE Center for Global Medicine, Seoul National University College of Medicine, Seoul, Korea

**Keywords:** Cancer, Korea, Guideline

## Abstract

**OBJECTIVES:**

The purpose of this study was to explore the association between adherence to 2 cancer prevention recommendations and cancer risk.

**METHODS:**

In total, 104,386 individuals aged 40-69 years old who were recruited between 2004 and 2013 in the Health Examinees-Gem study were included. Adherence scores were constructed based on 8 items from the World Cancer Research Fund/American Institute for Cancer Research (WCRF/AICR) cancer prevention recommendations, including body weight, physical activity, diet, alcohol consumption and breastfeeding, and on 6 items from the Korean cancer prevention guidelines (smoking status, eating vegetables and fruits, salty foods, alcohol intake, physical activity, and body weight). A Cox proportional hazards model was used to estimate the associations between adherence scores and the risk of total and 5 major cancers.

**RESULTS:**

The multivariable hazard ratio (HR) for total cancer with the high adherence score versus the lowest score (4.25-7.00 vs. 0.00-3.25) for the WCRF/AICR guidelines was 0.91 (95% confidence interval [CI], 0.82 to 1.00) in men. A reduced breast cancer risk was observed among women with the highest score. Men within the highest category of the Korean cancer prevention guideline score (3.25-6.00) had an HR of 0.80 (95% CI, 0.73 to 0.88) for developing total cancer compared to men within the lowest score (0.00-2.50). The higher adherence group among men showed lower risks of developing stomach, colorectal, and lung cancers.

**CONCLUSIONS:**

Adhering to guidelines for cancer prevention can help to reduce the risk of developing cancer in Korean men. The association might differ by cancer type and gender.

## GRAPHICAL ABSTRACT

[Fig f2-epih-45-e2023070]

## INTRODUCTION

Previous studies have shown that many aspects of cancer pathogenesis are related to lifestyle factors, including dietary habits [1]. It has also been reported that 30-50% of cancer cases worldwide can be prevented [2]. Countries and organizations around the world have published guidelines for cancer prevention. Among them, the recommendations for cancer prevention were updated in 2018 by the World Cancer Research Fund/American Institute for Cancer Research (WCRF/AICR) based on a comprehensive literature review by expert panels [1]. Other examples are the American Cancer Society guidelines [3] in the United States, the European Code [4] in Europe, 6 cancer prevention recommendations proposed in Japan [5]. and 10 cancer prevention recommendations in the Korea [6].

In 2018, the WCRF/AICR updated their cancer prevention guidelines, revising the recommendations made in 2007 [1]. The revised guidelines consist of 10 key points: (1) maintain a healthy weight; (2) engage in regular physical activity; (3) eat a better diet; (4) limit “fast foods”; (5) limit “consumption of red and processed meats”; (6) limit alcohol consumption; (7) cut down on sugary drinks; (8) breastfeed one’s baby if possible; (9) avoid using supplements; and (10) adhere to recommendations after a cancer diagnosis. These guidelines primarily focus on dietary habits, nutrition, and physical activity. However, they do not address other significant modifiable factors such as cigarette smoking or sun exposure. In Korea, national cancer prevention regulations and practice guidelines were established and disseminated in 2006, in line with the Cancer Prevention and Management Act, and were subsequently updated in 2016 [6]. According to the literature, there are 10 key factors for cancer prevention: smoking, consumption of vegetables and fruits, eat food without, alcohol consumption, physical activity, weight management, vaccination, sexual health, occupational safety, and cancer screening. Of these 10 factors, 4 are related to dietary habits, compared to the 6 dietary-related factors in the WCRF/AICR guidelines. Notably, the Korean guidelines include smoking, which is absent from the WCRF/AICR guidelines. Therefore, it would be intriguing to investigate the association between cancer risk and these 2 sets of guidelines within the same population.

A previous study found that strict adherence to the 2018 WCRF/AICR cancer prevention recommendations reduced the risk of total cancer [7] and mortality [8]. An inverse association between the WCRF/AICR cancer prevention recommendations adherence score and the incidence of cancer was reported for several cancers, including colorectal cancer [9-11], pancreatic cancer [12], prostate cancer [13] and breast cancer [14-16]. However, most of these studies were conducted in Western countries, such as the United States and European nations, and no studies have investigated these issues in Asia. In Korea, prospective studies have been conducted on individual factors of cancer prevention recommendations, such as cigarette smoking, alcohol consumption, and obesity, in relation to cancer risk [17]; however, none of those studies comprehensively addressed guideline adherence as a whole.

Therefore, our study aimed to determine whether adherence to the Korean cancer prevention guidelines and WCRF/AICR cancer prevention recommendations is associated with total cancer risk and the risk for 5 major cancers (lung, stomach, colorectal, breast, and prostate cancer) in the Korean population through a large-scale, population-based, prospective cohort study.

## MATERIALS AND METHODS

### Study population

The Health Examinees Study, a component of the Korean Genome and Epidemiology Study (KoGES) funded by the Korea Disease Control and Prevention Agency, is a large-scale population-based prospective cohort study aimed at identifying environmental and genetic factors for major chronic diseases. From 2004 to 2013, a total of 173,202 men and women were recruited from 38 hospitals and regional health check-up centers in 8 regions across Korea, selected according to a rigorous standardized research protocol. Details about the study design can be found in previous publications [18,19].

**Figure 1.**
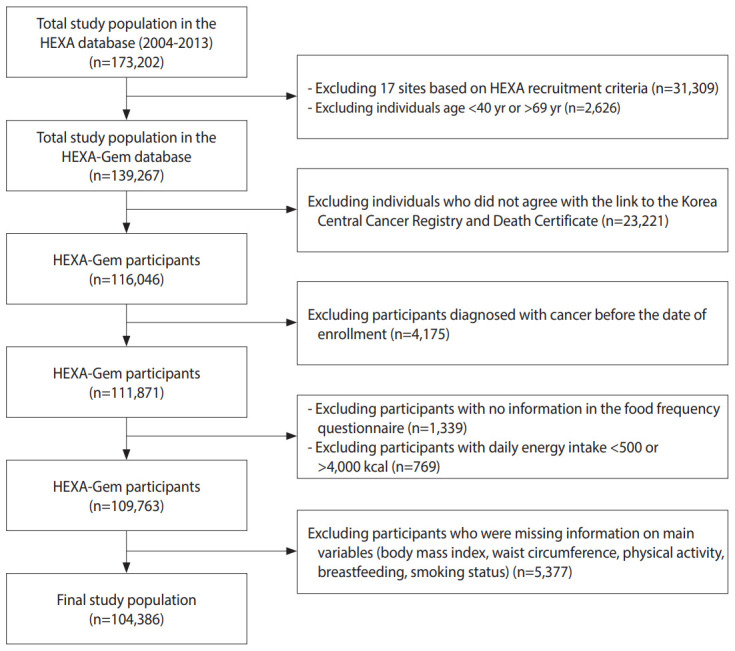
Flow chart of selection of study participants. HEXA, Health Examinees; HEXA-Gem, Health Examinees-Gem.

The Health Examinees-Gem (HEXA-G) cohort was created by applying additional qualification criteria for participating organizations [20]. Of the 139,267 participants aged 40-69 years old in the HEXA-G cohort, 34,881 were excluded for the following reasons: (1) people who disagreed with linkage between the Korea Central Cancer Registry and death certificates (n=23,221); (2) people diagnosed with cancer before the date of enrollment (n=4,175); (3) those without food frequency questionnaire information (n=1,339); (4) people with daily energy intake < 500 kcal or > 4,000 kcal (n=769); and (5) people who had missing data on the main variables (body mass index [BMI], waist circumference, physical activity, breastfeeding, and smoking status) (n=5,377). The final analysis included 104,386 participants, consisting of 36,266 men and 68,130 women (Figure 1).

### World Cancer Research Fund/American Institute for Cancer Research score construction

Information including participants’ general demographic characteristics, physical activity, lifestyle habits, and medical history was collected at baseline using structured questionnaires. For dietary information, an individual’s usual eating habits were estimated through the 106-food item semiquantitative food frequency questionnaire, which has been tested for validity and reliability [21]. Each nutrient intake and daily energy intake were calculated by the food composition table developed by the Korea Health Industry Development Institute [22].

A standardized scoring system developed by the National Cancer Institute (NCI) of the United States and members of the AICR and WCRF International team, based on the 2018 version of the WCRF/AICR cancer prevention recommendations, was used to facilitate international comparisons between study results [23]. Eight items, excluding supplement use and recommendations for cancer survivors, constituted the proposed 2018 WCRF/AICR cancer prevention recommendations score: (1) maintain a healthy weight; (2) engage in regular physical activity; (3) eat a better diet; (4) limit fast food; (5) limit “consumption of red and processed meats”; (6) cut down on sugary drinks; (7) limit alcohol consumption; and (8) breastfeed if possible. One point was assigned to complete adherence to each recommendation, 0.5 points for partial adherence, and 0.0 points for non-adherence [23]. When there were 2 subitems, such as the healthy weight item, the score was equally divided to maintain a total of 1.0 point. According to the guidelines of the Steering Committee of the Regional Office for the Western Pacific Region of the World Health Organization [24], the BMI criterion was assigned 0.50 points if it was satisfied (18.5-22.9 kg/m^2^), 0.25 points if it was partially satisfied (23.0-24.9 kg/m^2^), and 0.00 points if it was not satisfied (< 18.5 or > 25.0 kg/m^2^). The waist circumference score was based on the criteria of the Korean Society for the Study of Obesity; 0.50 points were assigned if the criterion was met (men: < 90 cm, women: < 80 cm), and 0.0 points was assigned if it was not met (men: ≥ 90 cm, women: ≥ 80 cm). Details of each score component are presented in Supplementary Material 1.

### Korean cancer prevention guidelines score construction

Among the 10 cancer prevention guidelines for Koreans, 6 items (smoking, vegetables and fruits, salty foods, alcohol, physical activity, and weight) were included in the score. For smoking, a score of 1.0 was assigned to never smokers, 0.5 to former smokers, and 0.0 to current smokers. For salty food consumption, the 2020 Dietary Reference Intakes for Koreans was used as a guide. A score of 1.0 was given for sodium intake less than 1,500 mg/day, 0.5 for intake between 1,500 mg/day and 2,300 mg/day, and 0.0 for intake of 2,300 mg/day or more [25]. The scoring for the remaining items was conducted in the same manner as the 2018 WCRF/AICR cancer prevention recommendations. Consequently, the total score for the 2018 WCRF/AICR cancer prevention recommendations, which is the aggregate of the scores for each recommendation, ranged from 0.0 points to 7.0 points for men and 0.0 points to 8.0 points for women. The Korean cancer prevention guideline score, however, had a maximum of 6.0 points for both genders. A higher score signifies greater adherence to cancer prevention recommendations. More details can be found in Supplementary Material 2.

### Ascertainment of cancer cases

Cancer cases were ascertained through data linkage from the Korea Central Cancer Registry, which has been supervising nationwide cancer registration since 1980 under the guidance of the Ministry of Health and Welfare. Incident cases were classified as those diagnosed with cancer after the baseline survey and up until December 31, 2018.

The top 5 major cancers were selected according to the Annual Report of Cancer Statistics in Korea in 2019 [26]. The most common cancer among Korean men and women was thyroid cancer, followed by lung cancer, stomach cancer, colorectal cancer, breast cancer, and prostate cancer. However, thyroid cancer was omitted from our selection due to the lack of identifiable preventable risk factors in comparison to the other cancers [1,27].

Incident cancer cases were identified using the 10th revision of the International Classification of Disease, 10th revision codes C33-C34 (lung cancer), C16 (stomach cancer), C18-C20 (colorectal cancer), C50 (breast cancer), and C61 (prostate cancer).

### Statistical analysis

Tertile score groups were used, considering the distribution of the adherence scores and examples from the previous literature [7,10,12,14,28-30]. The lowest group was analyzed as the reference group. Because the breastfeeding category only applied to women, all analyses were stratified by gender.

Participants’ characteristics by cancer prevention recommendation score category and gender are summarized using percentages for categorical variables and means and standard deviations for continuous variables. General characteristics according to cancer prevention recommendation scores were compared using the chi-square test for categorical variables and a generalized linear model for continuous variables. The median score of each category of cancer prevention guidelines was used as a continuous variable to test for trends. The proportional hazards assumption was tested using the Schoenfeld residuals method, and no evidence of violating the assumption was found (p>0.05 for all). A Cox proportional hazards regression model was used to evaluate the associations of cancer risk with WCRF/AICR cancer prevention recommendation scores and Korean cancer prevention guideline scores. The results are presented as hazard ratios (HRs) and 95% confidence intervals (CIs). Age was chosen as the time scale [31]. The entry time was age at baseline when recruiting cohorts, and the exit time was age at the date of diagnosis of cancer, death, or the last date of follow-up (December 31, 2018), whichever came first.

We adjusted for potential confounding variables, including education level (categorized as less than high school, high school, college or more, and missing), smoking status (divided into never smoker, ex-smoker, current smoker, and missing), total energy intake (grouped into tertiles), and family history of cancer (classified as yes, no, or missing). These variables were chosen based on previous literature. Missing data in categorical covariates were included in the multivariable Cox proportional hazards regression models as a dummy category. We estimated individual associations for each component of the WCRF/AICR cancer prevention recommendations score and the Korean Cancer Prevention Guidelines score in relation to cancer risk. To determine risk estimates and 95% CIs per 1-point increment, the WCRF/AICR cancer prevention recommendations scores and the Korean Cancer Prevention Guidelines score were also modeled as direct continuous variables. To exclude potential reverse causation due to the prevalent cancers not diagnosed at baseline, a sensitivity analysis was conducted excluding the initial 2 years of follow-up. All analyses were carried out using SAS version 9.4 (SAS Institute Inc., Cary, NC, USA).

### Ethics statement

The present study guidelines were approved by the Institutional Review Boards of the Seoul National University Hospital in Seoul, Korea (IRB No. E-2212-006-1381), and the Ethics Committee of the KoGES of the Korea National Institute of Health (IRB No. 2014-08-02-3C-A). After providing a detailed description of the study, prior written consent was collected from all participants.

## RESULTS

Table 1 shows the general characteristics of the participants according to adherence to the WCRF/AICR cancer prevention recommendations. Those with high adherence tended to be older, engage in more physical activity, and consume more fruits and vegetables compared to those with low adherence. Both men and women with high adherence had lower BMI, alcohol consumption, red meat consumption, and total energy intake. The baseline characteristics for adherence to the Korean cancer prevention guidelines were largely similar to those for the WCRF/AICR cancer prevention recommendations. However, in contrast to the WCRF/AICR cancer prevention recommendations, those in the highest tertiles for adherence to the Korean cancer prevention guidelines had higher education and income levels (Supplementary Material 3).

During a median follow-up period of 9.04 years, we identified a total of 6,987 cancer cases, comprised of 2,758 men and 4,229 women. Tables 2 and 3 display the risk of cancer in relation to the adherence score of the WCRF/AICR cancer prevention recommendation. In models adjusted for confounding factors, a high adherence score to the WCRF/AICR cancer prevention recommendations was associated with a 9% reduction in total cancer risk (HR, 0.91; 95% CI, 0.82 to 1.00) in men. A high adherence score to the Korean cancer prevention guidelines was associated with a reduced risk of total cancer (HR, 0.80; 95% CI, 0.73 to 0.88), stomach cancer (HR, 0.68; 95% CI, 0.54 to 0.84), colorectal cancer (HR, 0.74; 95% CI, 0.58 to 0.95), and lung cancer (HR, 0.37; 95% CI, 0.27 to 0.51) in men. In women, no association was found between total cancer and other types of cancer, but an inverse association was observed with breast cancer (HR, 0.80; 95% CI, 0.67 to 0.95). However, for stomach cancer in women, the risk increased by 13% for each 1-point increase in the WCRF/AICR cancer prevention recommendation score (HR, 1.13; 95% CI, 1.02 to 1.25)

Table 4 and Supplementary Materials 4 and 5 present the associations between cancer risk and adherence to the individual components of the cancer prevention guideline score. The group with normal weight exhibited a 12% (HR, 0.88; 95% CI, 0.81 to 0.97) reduction in total cancer risk for men and a 10% (HR, 0.90; 95% CI, 0.83 to 0.96) reduction for women, compared to the underweight or obese group. Participants who met the optimal criteria for abdominal obesity (men: < 90 cm, women: < 80 cm) demonstrated a 13% (HR, 0.87; 95% CI, 0.81 to 0.95) decrease in total cancer risk for men and a 12% (HR, 0.88; 95% CI, 0.81 to 0.94) decrease for women, compared to those with abdominal obesity (men: ≥ 90 cm, women: ≥ 80 cm). Our findings also revealed that men who have never smoked had a 27% (HR, 0.73; 95% CI, 0.66 to 0.81) reduction in total cancer risk compared to current smokers.

In a sensitivity analysis, we examined data from 35,659 men and 67,112 women, excluding the first 2 years of follow-up (Supplementary Materials 6 and 7). The findings were generally similar to the results from the main analysis. In the main analysis, women who scored highly on the WCRF/AICR cancer prevention recommendation adherence scale demonstrated a 20% decrease in breast cancer risk. However, this association was not significant in the sensitivity analysis. Conversely, women who scored highly on the Korean cancer prevention guideline scale exhibited a 25% decrease in colorectal cancer risk in the sensitivity analysis (HR, 0.75; 95% CI, 0.57 to 0.98).

## DISCUSSION

In this large-scale, prospective cohort study involving the Korean population, we found an inverse association between total cancer incidence and adherence to the WCRF/AICR cancer prevention guidelines among men. The Korean cancer prevention guidelines also showed an inverse association with adherence scores for total cancer, stomach cancer, colorectal cancer, and lung cancer. In women, only breast cancer showed an inverse association with adherence to the WCRF/AICR cancer prevention recommendations.

No studies have yet explored the association between cancer risk and adherence to the 2007 or 2018 versions of the WCRF/AICR cancer prevention recommendations in Asian countries. Furthermore, the effectiveness of Korean cancer prevention guidelines remains unexamined. However, research has been conducted on the association between adherence to the 2007 version of the WCRF/AICR cancer prevention recommendations and cancer risk within the European population [7,32]. Despite variations in scoring systems, items, and racial and population characteristics, numerous studies have consistently reported that adherence to cancer prevention guidelines can help prevent cancer. Our findings align with these studies. Higher adherence to the WCRF/AICR cancer prevention recommendations was associated with a reduced risk of total cancer in the United States [33] and Sweden [7], breast cancer in Europe [14,16] and South Africa [15], and colorectal cancer in Spain [9] and the United States [10,11].

Among the individual factors of the WCRF/AICR cancer prevention recommendations and Korean cancer prevention guidelines, strong adherence to weight management and smoking was associated with a decrease in cancer risk. Previous studies have identified obesity [34] and smoking [35] as significant risk factors among many for various types of cancer. Aside from obesity and smoking, no significant association was found with cancer risk with other individual components of the cancer prevention recommendation score.

There were no observed associations between adherence to Korean cancer prevention guidelines and total cancers and 5 types of cancer in women. Similarly, no associations were found with other types of cancer in the WCRF/AICR cancer prevention recommendations, with the exception of breast cancer. Case-control studies conducted in South Africa [15] and Italy [16] and a cohort study in Spain [14], found that a higher score on the 2018 WCRF/AICR cancer prevention recommendations was associated with a lower risk of breast cancer. This finding was corroborated by a meta-analysis [16]. Our study discovered that for women, each one-point increase in the WCRF/AICR cancer prevention recommendations score corresponded to a 13% higher risk of stomach cancer. The precise reason for this result remains unclear, but it is plausible that the high rate of *Helicobacter pylori* infection, which is not included in the WCRF/AICR cancer prevention recommendations, played a significant role. The International Agency for Research on Cancer (IARC) classifies *H. pylori* as a class 1 carcinogen [36]. The population-attributable fraction of *H. pylori* infection for stomach cancer was 80.3% for men and 78.7% for women in Korea [37], which is higher than in China (63%) and in European countries (20-30%) and similar to that in Japan [38]. The main risk factors for gastric cancer among Korean women, such as age, BMI, having a family history of cancer, and past smoking status, are not adequately reflected in the WCRF/AICR recommendations [39]. In this study, we found that the risk of gastric cancer increased in women who scored highly on the individual components of breastfeeding and fast food restriction. As there are no other published studies for comparison, further research is required.

With the exception of breast cancer, our study found a reduced cancer risk only in men who adhered to the cancer prevention recommendations, while no association was found in women. A United States study similarly found a stronger inverse association for colorectal cancer in men than in women [11]. The mechanisms behind these gender differences remain unclear, but we present several potential explanations from various perspectives. One possibility is the involvement of gender hormones. Prior research has indicated that the use of exogenous hormones, such as postmenopausal hormone therapy and oral contraceptives, as well as the increase in female hormones due to pregnancy and childbirth, may help prevent the development of colorectal cancer in women [40-42]. Another potential explanation could be gender-based differences in dietary intake and eating behavior. In Korean culture, women are more likely to purchase and prepare food, which may lead to greater nutritional knowledge and a tendency towards healthier dietary choices compared to men [43,44]. Furthermore, the number of current female smokers in our study population was extremely low (less than 5%), and the percentage of current female drinkers was 31.4%, with an average ethanol intake of 6.28 g/day. Both alcohol and smoking have been identified as risk factors for several types of cancer and are included in cancer prevention guidelines. This significant disparity in alcohol and smoking habits between men and women may have influenced our results.

Sodium is a crucial element for maintaining homeostasis and supporting various physiological functions in the body, and it is typically acquired through salt consumption [45]. Both insufficient and excessive sodium intake can lead to a range of clinical problems. Research indicates that when sodium intake drops below 700 mg/day, it is associated with elevated total cholesterol levels in the blood [46] and increased insulin resistance [47]. According to our data, 3.36% of men and 4.62% of women reported a sodium intake of less than 700 mg/day (data not shown). Some studies have suggested a connection between a preference for salty food, salt consumption, and stomach cancer [48,49], but currently, there is no precise numerical guideline for determining the appropriate sodium intake. The evidence linking low sodium intake to cancer is both scarce and difficult to establish. Consequently, the current cancer prevention recommendation aligns with the 2020 Dietary Reference Intakes for Koreans, suggesting a sodium intake of less than 1,500 mg/day. However, it is important to acknowledge that these guidelines may need to be revised as more evidence becomes available in the future.

Our study has several limitations that should be taken into account. First, due to the absence of data, 4 out of 10 Korean cancer prevention guidelines were not included. Further research in Korea is required to incorporate these missing elements, namely: vaccination, sexual health, safety and health, and cancer screening. Second, the cancer prevention guidelines used in our study, including the Korean cancer prevention guidelines and the WCRF/AICR cancer prevention recommendations, utilized a standardized scoring system for ease of comparison. This system did not assign weights to the components, assuming all to be of equal importance. Future studies should consider the relative weights of these components and their relevance to different racial groups. Thirdly, despite adjusting for confounding factors, it is possible that some residual and unmeasured confounding factors were not eliminated. Despite these limitations, this study is significant as it is a large-scale investigation into the associations between adherence to cancer prevention guidelines, including diet, and cancer risk, a topic that has primarily been studied in the Western world. Another strength of this study is that the accuracy of cancer diagnoses was ensured by linkage to the Korea Central Cancer Registry, which contained an estimated 98.3% of data on cancer incidence in 2019 [50].

In conclusion, these cohort study’s findings indicate that adherence to both the WCRF/AICR cancer prevention recommendations and Korean cancer prevention guidelines can prevent cancer in men. Additionally, we discovered that adherence to the WCRF/AICR cancer prevention recommendations can aid in preventing breast cancer in Korean women. The effectiveness of these guidelines may vary depending on the type of cancer and the individual’s gender. However, due to data limitations, we were unable to consider all aspects of the Korean cancer prevention guidelines. Therefore, further research is required.

## Figures and Tables

**Figure f2-epih-45-e2023070:**
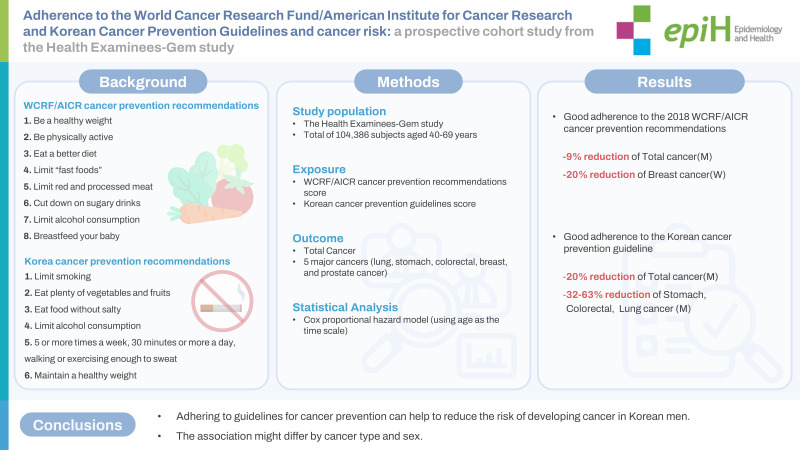


**Table 1.** Baseline characteristics of the participants according to WCRF/AICR cancer prevention adherence score categories and gender

Characteristics		WCRF/AICR adherence score							
		Men (n=36,266)				Women (n=68,130)			
		Tertile 1	Tertile 2	Tertile 3	p-value^1^	Tertile 1	Tertile 2	Tertile 3	p-value^1^
Score range		0.00≤score<3.25	3.25≤score<4.25	score≥4.25		0.00≤score<4.50	4.50≤score<5.50	score≥5.50	
No. of participants		10,947 (30.2)	13,091 (36.1)	12,228 (33.7)	<0.001	19,732 (29.0)	24,186 (35.5)	24,212 (35.5)	<0.001
Age (yr)		50.9±8.2	53.4±8.3	56.0±7.9	<0.001	49.3±7.5	52.5±7.7	54.5±7.2	<0.001
Body mass index (kg/m^2^)		25.5±2.9	24.4±2.7	23.5±2.2	<0.001	24.2±3.3	23.7±3.0	23.0±2.5	<0.001
Education					<0.001				<0.001
	≤Middle school	1,973 (18.0)	2,799 (21.4)	2,554 (20.9)		4,977 (25.2)	9,003 (37.2)	10,145 (41.9)	
	High school	4,579 (41.8)	5,301 (40.5)	4,928 (40.3)		9,018 (45.7)	10,233 (42.3)	10,336 (42.7)	
	≥College	4,309 (39.4)	4,881 (37.3)	4,623 (37.8)		5,614 (28.5)	4,784 (19.8)	3,552 (14.7)	
	Missing	86 (0.8)	110 (0.8)	123 (1.0)		123 (0.6)	166 (0.7)	179 (0.7)	
Income (10^4^ Korean won)					<0.001				<0.001
	<200	2,168 (19.8)	3,172 (24.2)	3,274 (26.8)		5,003 (25.3)	7,475 (30.9)	7,786 (32.2)	
	200-400	5,038 (46.0)	5,631 (43.0)	5,042 (41.2)		8,257 (41.9)	9,341 (38.6)	9,231 (38.1)	
	≥400	3,250 (29.7)	3,548 (27.1)	3,180 (26.0)		5,320 (27.0)	5,707 (23.6)	5,275 (21.8)	
	Missing	491 (4.5)	740 (5.7)	732 (6.0)		1,152 (5.8)	1,663 (6.9)	1,920 (7.9)	
Smoking status					<0.001				<0.001
	Never	2,366 (21.6)	3,485 (26.6)	3,940 (32.3)		18,505 (93.8)	23,428 (96.9)	23,755 (98.1)	
	Former	4,074 (37.2)	5,291 (40.4)	5,446 (44.5)		409 (2.0)	269 (1.1)	180 (0.8)	
	Current	4,507 (41.2)	4315 (33.0)	2,842 (23.2)		818 (4.2)	489 (2.0)	277 (1.1)	
	Missing								
Alcohol intake, (g of ethanol/day)		23.9±44.5	14.7±26.4	8.6±19.2	<0.001	3.8±13.5	1.7±26.1	0.7±4.1	<0.001
Physical activity (min/wk)					<0.001				<0.001
	<75	8,170(74.6)	6,635(50.7)	2,438(19.9)		15,863(80.3)	14,565(60.2)	6,016(24.9)	
	75-149	891 (8.2)	1,414 (10.8)	1,060 (8.7)		1,410 (7.2)	2,457 (10.2)	2,332 (9.6)	
	≥150	1,886 (17.2)	5,042 (38.5)	8,730 (71.4)		2,459 (12.5)	7,164 (29.6)	15,864 (65.5)	
Family history of cancer					0.618				0.615
	No	8,049 (73.5)	9,521 (72.7)	8,967 (73.3)		13,878 (70.3)	16,951 (70.1)	17,081 (70.5)	
	Yes	2,877 (26.3)	3,539 (27.1)	3,233 (26.5)		5,803 (29.4)	7,178 (29.7)	7,082 (29.3)	
	Missing	21 (0.2)	31 (0.2)	28 (0.2)		51 (0.3)	57 (0.2)	49 (0.2)	
Breastfeeding (mo)									<0.001
	None	-	-	-		7,987 (40.5)	3,629 (15.0)	1,083 (4.4)	
	<6	-	-	-		2,628 (13.3)	2,181 (9.0)	1,151 (4.8)	
	≥6	-	-	-		9,117 (46.2)	18,376 (76.0)	21,978 (90.8)	
Energy intake (kcal/day)		2,012.2±533.7	1,823.4±489.9	1,718.4±420.9	<0.001	1,809.5±544.4	1,667.2±503.6	1,613.2±454.8	<0.001
Vegetable and fruits intake (g/day)		244.5±163.1	260.5±178.4	299.3±191.7	<0.001	226.4±153	247±168.9	300.6±197.1	<0.001
Fast food intake (g/day)		61.7±51.7	40.8±44.5	21.9±29.8	<0.001	57.8±54.6	35.0±43.6	18.1±29.4	<0.001
Red meat intake (g/day)		78.5±58.3	49.8±48.2	33.6±31.9	<0.001	57.1±55.2	35.1±39.3	25.8±27.4	<0.001
Sugar-sweetened drinks (g/day)		75.0±104.7	54.8±78.3	47.2±62.1	<0.001	64.8±93.0	54.8±75.5	51.3±66.7	<0.001
Sodium intake (g/day)		2,637.7±1,371.2	2,587.4±1,432.0	2,717.5±1,450.8	<0.001	2,296.3±1,226.6	2,298.3±1,301.1	2,559.2±1,412.6	<0.001

Values are presented as number (%) or mean±standard deviation. WCRF/AICR, World Cancer Research Fund/American Institute for Cancer Research.

1 Chi-square test (for categorical variables) and generalized linear model (for continuous variables).

**Table 2.** HRs and 95% CIs for cancer risk according to WCRF/AICR cancer prevention guideline adherence score categories^1^

Variables		Men (n=36,266)					Women (n=68,130)				
		Tertile 1	Tertile 2	Tertile 3	p for trend^2^	Continuous (per 1-point increase in score)	Tertile 1	Tertile 2	Tertile 3	p for trend^2^	Continuous (per 1-point increase in score)
Score range		0.00≤score<3.25	3.25≤score<4.25	score≥4.25	-	-	0.00≤score<4.50	4.50≤score<5.50	score≥5.50	-	-
Person-years		96,520.70	115,445.60	107,406.40	-	-	172,867.90	214,532.00	215,386.00	-	-
Total cancer											
	No. of cases/total subjects	724/10,947	1,000/13,091	1,034/12,228	-	-	1,173/19,732	1,510/24,186	1,546/24,212	-	-
	Crude HR (95% CI)	1.00 (reference)	0.95 (0.86, 1.05)	0.89 (0.80, 0.98)	0.013	0.97 (0.93, 1.00)	1.00 (reference)	0.98 (0.91, 1.06)	0.97 (0.89, 1.05)	0.394	0.99 (0.96, 1.02)
	Multivariable-adjusted HR (95% CI)	1.00 (reference)	0.96 (0.87, 1.05)	0.91 (0.82, 1.00)	0.053	0.98 (0.94, 1.02)	1.00 (reference)	0.99 (0.91, 1.07)	0.98 (0.90, 1.06)	0.553	0.99 (0.96, 1.02)
Stomach cancer											
	No. of cases/total subjects	134/10,947	183/13,091	181/12,228	-	-	87/19,732	137/24,186	178/24,212	-	-
	Crude HR (95% CI)	1.00 (reference)	0.97 (0.77, 1.21)	0.89 (0.71, 1.12)	0.314	0.95 (0.87, 1.04)	1.00 (reference)	1.09 (0.83, 1.43)	1.29 (0.99, 1.69)	0.050	1.14 (1.04, 1.26)
	Multivariable-adjusted HR (95% CI)	1.00 (reference)	1.00 (0.79, 1.25)	0.95 (0.75, 1.21)	0.651	0.98 (0.89, 1.08)	1.00 (reference)	1.07 (0.81, 1.41)	1.26 (0.96, 1.65)	0.080	1.13 (1.02, 1.25)
Colorectal cancer											
	No. of cases/total subjects	111/10,947	143/13,091	143/12,228	-	-	89/19,732	152/24,186	147/24,212	-	-
	Crude HR (95% CI)	1.00 (reference)	0.92 (0.72, 1.18)	0.87 (0.67, 1.11)	0.260	0.93 (0.84, 1.03)	1.00 (reference)	1.12 (0.86, 1.46)	0.97 (0.74, 1.27)	0.742	0.97 (0.88, 1.07)
	Multivariable-adjusted HR (95% CI)	1.00 (reference)	0.90 (0.70, 1.16)	0.83 (0.64, 1.08)	0.161	0.91 (0.82, 1.02)	1.00 (reference)	1.14 (0.87, 1.49)	0.99 (0.76, 1.31)	0.864	0.98 (0.89, 1.08)
Lung cancer											
	No. of cases/total subjects	76/10,947	114/13,091	101/12,228	-	-	62/19,732	93/24,186	88/24,212	-	-
	Crude HR (95% CI)	1.00 (reference)	0.95 (0.71, 1.27)	0.70 (0.52, 0.95)	0.015	0.90 (0.81, 1.00)	1.00 (reference)	0.89 (0.65, 1.23)	0.73 (0.53, 1.01)	0.047	0.91 (0.80, 1.03)
	Multivariable-adjusted HR (95% CI)	1.00 (reference)	1.02 (0.76, 1.37)	0.86 (0.63, 1.18)	0.333	0.99 (0.88, 1.11)	1.00 (reference)	0.94 (0.68, 1.30)	0.77 (0.55, 1.07)	0.102	0.93 (0.82, 1.06)
Prostate (men)/Breast cancer (women)											
	No. of cases/total subjects	103/10,947	153/13,091	193/12,228	-	-	294/19,732	295/24,186	273/24,212	-	-
	Crude HR (95% CI)	1.00 (reference)	0.89 (0.70, 1.14)	0.91 (0.72, 1.16)	0.507	0.97 (0.88, 1.07)	1.00 (reference)	0.84 (0.71, 0.98)	0.79 (0.66, 0.94)	0.006	0.91 (0.85, 0.97)
	Multivariable-adjusted HR (95% CI)	1.00 (reference)	0.89 (0.69, 1.14)	0.89 (0.70, 1.13)	0.367	0.95 (0.87, 1.05)	1.00 (reference)	0.84 (0.71, 1.00)	0.80 (0.67, 0.95)	0.010	0.91 (0.85, 0.97)

HR, hazard ratio; CI, confidence interval; WCRF/AICR, World Cancer Research Fund/American Institute for Cancer Research.

1 Adjusted for education level (less than high school, high school, college or above, and missing), smoking status (non-smoker, ex-smoker, current smoker, and missing), total energy intake (tertiles), and family history of cancer (yes, no, and missing).

2 The test for trend was calculated with the median score for each category of the cancer prevention guideline as a continuous variable.

**Table 3.** Hazard ratios (HRs) and 95% confidence intervals (CIs) for cancer risk according to Korean cancer prevention guideline adherence score categories^1^

Variables		Men (n=36,266)					Women (n=68,130)				
		Tertile 1	Tertile 2	Tertile 3	p for trend^2^	Continuous (per 1-point increase in score)	Tertile 1	Tertile 2	Tertile 3	p for trend^2^	Continuous (per 1-point increase in score)
Score range		0.00≤score<2.50	2.50≤score<3.25	score≥3.25	-	-	0.00≤score<3.50	3.50≤score<4.00	score≥4.00	-	-
Person-years		101,000.20	106,238.80	112,133.70	-	-	194,374.80	151,862.50	256,548.60	-	-
Total cancer											
	No. of cases/total subjects	827/11,402	975/12,139	956/12,725	-	-	1,396/21,979	1,031/17,065	1,802/29,086	-	-
	Crude HR (95% CI)	1.00 (reference)	0.97 (0.88, 1.06)	0.80 (0.73, 0.88)	<0.001	0.90 (0.86, 0.94)	1.00 (reference)	0.95 (0.88, 1.03)	0.97 (0.91, 1.05)	0.587	0.98 (0.95, 1.02)
	Multivariable-adjusted HR (95% CI)	1.00 (reference)	0.97 (0.88, 1.06)	0.80 (0.73, 0.88)	<0.001	0.90 (0.86, 0.94)	1.00 (reference)	0.94 (0.87, 1.02)	0.96 (0.90, 1.03)	0.407	0.98 (0.94, 1.02)
Stomach cancer											
	No. of cases/total subjects	168/11,402	171/12,139	159/12,725	-	-	132/21,979	80/17,065	190/29,086	-	-
	Crude HR (95% CI)	1.00 (reference)	0.85 (0.69, 1.06)	0.68 (0.55, 0.85)	<0.001	0.83 (0.75, 0.92)	1.00 (reference)	0.79 (0.60, 1.04)	1.09 (0.88, 1.36)	0.253	1.07 (0.94, 1.22)
	Multivariable-adjusted HR (95% CI)	1.00 (reference)	0.85 (0.69, 1.05)	0.68 (0.54, 0.84)	<0.001	0.83 (0.75, 0.92)	1.00 (reference)	0.79 (0.59, 1.04)	1.09 (0.87, 1.36)	0.276	1.07 (0.94, 1.22)
Colorectal cancer											
	No. of cases/total subjects	134/11,402	128/12,139	135/12,725	-	-	132/21,979	98/17,065	158/29,086	-	-
	Crude HR (95% CI)	1.00 (reference)	0.81 (0.64, 1.04)	0.74 (0.58, 0.94)	0.021	0.90 (0.81, 1.01)	1.00 (reference)	0.97 (0.74, 1.25)	0.89 (0.71, 1.13)	0.328	0.93 (0.81, 1.06)
	Multivariable-adjusted HR (95% CI)	1.00 (reference)	0.81 (0.64, 1.04)	0.74 (0.58, 0.95)	0.047	0.90 (0.81, 1.01)	1.00 (reference)	0.95 (0.73, 1.24)	0.88 (0.69, 1.11)	0.254	0.92 (0.80, 1.05)
Lung cancer											
	No. of cases/total subjects	111/11,402	114/12,139	66/12,725	-	-	89/21,979	48/17,065	106/29,086	-	-
	Crude HR (95% CI)	1.00 (reference)	0.79 (0.60, 1.02)	0.36 (0.27, 0.49)	<0.001	0.68 (0.60, 0.76)	1.00 (reference)	0.72 (0.50, 1.02)	0.92 (0.69, 1.21)	0.744	1.03 (0.86, 1.23)
	Multivariable-adjusted HR (95% CI)	1.00 (reference)	0.80 (0.61, 1.04)	0.37 (0.27, 0.51)	<0.001	0.69 (0.61, 0.78)	1.00 (reference)	0.70 (0.49, 0.99)	0.88 (0.66, 1.17)	0.554	1.01 (0.84, 1.20)
Prostate (men)/Breast cancer (women)											
	No. of cases/total subjects	95/11,402	154/12,139	200/12,725	-	-	289/21,979	218/17,065	355/29,086	-	-
	Crude HR (95% CI)	1.00 (reference)	1.20 (0.93, 1.56)	1.22 (0.96, 1.56)	0.154	1.07 (0.96, 1.19)	1.00 (reference)	0.96 (0.81, 1.15)	0.93 (0.80, 1.09)	0.389	0.95 (0.87, 1.04)
	Multivariable-adjusted HR (95% CI)	1.00 (reference)	1.18 (0.92, 1.53)	1.18 (0.92, 1.51)	0.260	1.05 (0.95, 1.17)	1.00 (reference)	0.93 (0.78, 1.11)	0.88 (0.75, 1.04)	0.132	0.92 (0.84, 1.01)

1 Adjusted for education level (less than high school, high school, college or above, and missing), total energy intake (tertiles), and family history of cancer (yes, no, and missing).

2 The test for trend was calculated with the median score for each category of the cancer prevention guideline as a continuous variable.

**Table 4.** Associations between adherence to individual components of the 2 cancer prevention guideline scores and total cancer risk^1^

Components of the cancer prevention guideline score		Men (n=36,266)				Women (n=68,130)			
		No. of cases/total participants	Person-year	Crude HR (95% CI)	Multivariable-adjusted HR (95% CI)	No. of cases/total participants	Person-year	Crude HR (95% CI)	Multivariable-adjusted HR (95% CI)
Maintain a healthy weight (BMI)^2,3^									
	0.00	1,198/15,038	132,232.10	1.00 (reference)	1.00 (reference)	1,369/20,481	180,210.80	1.00 (reference)	1.00 (reference)
	0.25	792/10,966	96,903.70	0.86 (0.79, 0.94)	0.86 (0.79, 0.94)	1,147/18,088	161,030.00	0.95 (0.88, 1.03)	0.95 (0.88, 1.03)
	0.50	768/10,262	90,236.90	0.89 (0.82, 0.98)	0.88 (0.81, 0.97)	1,713/29,561	261,545.10	0.91 (0.85, 0.98)	0.90 (0.83, 0.96)
Maintain a healthy weight (WC)^2,3^									
	0.0	921/10,506	92,990.00	1.00 (reference)	1.00 (reference)	1,003/13,984	124,309.40	1.00 (reference)	1.00 (reference)
	0.5	1,837/25,760	226,382.70	0.87 (0.81, 0.94)	0.87 (0.81, 0.95)	3,226/54,146	478,476.50	0.89 (0.83, 0.96)	0.88 (0.81, 0.94)
Be physically active^2,3^									
	0.0	1,293/17,243	153,028.70	1.00 (reference)	1.00 (reference)	2,232/36,444	324,485.80	1.00 (reference)	1.00 (reference)
	0.5	229/3,365	30,058.30	0.97 (0.85, 1.12)	1.01 (0.87, 1.16)	386/6,199	55,514.50	1.01 (0.91, 1.13)	1.00 (0.90, 1.12)
	1.0	1,236/15,658	136,285.80	0.94 (0.87, 1.02)	0.98 (0.90, 1.06)	1,611/25,487	222,785.50	1.04 (0.97, 1.11)	1.03 (0.97, 1.10)
Eat a better diet^2,3^									
	0.0	1,115/14,175	124,235.30	1.00 (reference)	1.00 (reference)	1,762/28,614	250,984.80	1.00 (reference)	1.00 (reference)
	0.5	1,203/16,220	142,456.70	0.96 (0.88, 1.04)	0.96 (0.88, 1.04)	1,849/29,557	260,334.70	1.01 (0.95, 1.08)	1.01 (0.94, 1.08)
	1.0	440/5,871	52,680.80	0.96 (0.86, 1.08)	0.97 (0.87, 1.10)	618/9,959	91,466.30	0.96 (0.88, 1.05)	0.95 (0.87, 1.05)
Limit fast foods^2^									
	0.0	719/12,078	106,275.40	1.00 (reference)	1.00 (reference)	1,329/22,675	200,205.80	1.00 (reference)	1.00(reference)
	0.5	927/12,158	106,979.00	1.09 (0.99, 1.20)	1.08 (0.98, 1.20)	1,400/22808	201,475.80	1.00 (0.93, 1.08)	1.01 (0.94, 1.10)
	1.0	1,112/12,030	106,118.40	1.05 (0.95, 1.15)	1.04 (0.94, 1.16)	1,500/22,647	201,104.30	1.01 (0.94, 1.09)	1.04 (0.96, 1.13)
Limit red and processed meat^2^									
	0.0	580/8,857	78,183.60	1.00 (reference)	1.00 (reference)	587/9,901	86,949.20	1.00 (reference)	1.00 (reference)
	0.5	83/1,975	17,114.30	0.85 (0.68, 1.07)	0.85 (0.67, 1.07)	194/3,643	31,001.70	0.99 (0.84, 1.17)	0.99 (0.84, 1.16)
	1.0	2,095/25,434	224,074.90	1.04 (0.95, 1.14)	1.05 (0.95, 1.16)	3,448/54,586	484,835.00	1.01 (0.92, 1.10)	1.01 (0.92, 1.11)
Cut down on sugary drinks^2^									
	0.0	97/1,393	12,506.20	1.00 (reference)	1.00 (reference)	137/2,303	21,171.20	1.00 (reference)	1.00 (reference)
	0.5	2,601/34,352	302,363.00	0.92 (0.75, 1.13)	0.90 (0.73, 1.11)	4,043/64,961	573,944.50	1.07 (0.90, 1.27)	1.08 (0.91, 1.29)
	1.0	60/521	4,503.50	1.22 (0.88, 1.69)	1.18 (0.85, 1.64)	49/866	7,670.20	0.93 (0.67, 1.29)	0.96 (0.69, 1.33)
Limit alcohol consumption^2,3^									
	0.0	469/6,442	56,408.00	1.00 (reference)	1.00 (reference)	120/2,224	19,311.50	1.00 (reference)	1.00 (reference)
	0.5	1,405/19,788	174,640.80	0.88 (0.79, 0.97)	0.91 (0.81, 1.01)	1,063/18,455	162,073.20	1.04 (0.86, 1.26)	1.04 (0.86, 1.26)
	1.0	884/10,036	88,323.90	0.93 (0.83, 1.04)	0.99 (0.88, 1.11)	3,046/47,451	421,401.20	1.07 (0.89, 1.29)	1.07 (0.89, 1.30)
For mothers: breastfeed your baby^2^									
	0.0	-	-	-	-	762/12,699	110,432.50	1.00 (reference)	1.00 (reference)
	0.5	-	-	-	-	319/5,960	52,663.30	0.90 (0.79, 1.03)	0.90 (0.79, 1.02)
	1.0	-	-	-	-	3,148/49,471	439,690.10	0.96 (0.88, 1.04)	0.98 (0.90, 1.07)
Limit smoking^3,4^									
	0.0	864/11,664	102,415.50	1.00 (reference)	1.00 (reference)	94/1,584	13,646.70	1.00 (reference)	1.00 (reference)
	0.5	1,191/14,811	129,113.40	0.81 (0.74, 0.89)	0.81 (0.74, 0.89)	53/858	7,503.90	1.01 (0.72, 1.41)	1.00 (0.71, 1.39)
	1.0	703/9,791	87,843.80	0.73 (0.66, 0.80)	0.73 (0.66, 0.81)	4,082/65,688	581,635.30	0.97 (0.79, 1.19)	0.96 (0.78, 1.18)
Eat food without salty (sodium intake)^3^									
	0.0	1,470/20,060	178,067.60	1.00 (reference)	1.00 (reference)	2,010/31,956	157,922.30	1.00 (reference)	1.00 (reference)
	0.5	675/8,784	76,092.30	1.03 (0.94, 1.13)	1.03 (0.94, 1.13)	1,090/18,236	286,165.80	0.98 (0.91, 1.05)	0.98 (0.91, 1.06)
	1.0	613/7,422	65,212.80	1.04 (0.95, 1.14)	1.04 (0.94, 1.16)	1,129/17,938	158,697.80	1.01 (0.94, 1.08)	1.01 (0.94, 1.10)

HR, hazard ratio; CI, confidence interval; BMI, body mass index; WC, waist circumference; WCRF/AICR, World Cancer Research Fund/American Institute for Cancer Research.

1 Adjusted for education level (less than high school, high school, college or above, and missing), smoking status (non-smoker, ex-smoker, current smoker, and missing), total energy intake (tertiles), and family history of cancer (yes, no, and missing).

2 Components of the WCRF/AICR score.

3 Components of the Korean Cancer Prevention Guidelines score.

4 Adjusted for education level (less than high school, high school, college or above, and missing), total energy intake (tertiles), and family history of cancer (yes, no, and missing).

## SUPPLEMENTARY MATERIALS

Supplement Material 1. Operationalization of the WCRF/AICR cancer prevention recommendations in the HEXA study. N (%)

Supplement Material 2. Operationalization of the Korea cancer prevention recommendations in the HEXA study. N (%)

Supplement Material 3. Baseline characteristics of the participants according to Korean cancer prevention guideline categories and gender.

Supplement Material 4. Associations between adherence to individual components of the 2 cancer prevention guideline score and cancer risk in men.

Supplement Material 5. Associations between adherence to individual components of the 2 cancer prevention guideline score and cancer risk in women.

Supplement Material 6. Hazard ratios (HRs) and 95% confidence intervals (CI) for sensitivity analysis of cancer risk according to WCRF/AICR cancer prevention guideline adherence score categories^1^

Supplement Material 7. Hazard ratios (HRs) and 95% confidence intervals (CI) for sensitivity analysis of cancer risk according to Korean cancer prevention guideline adherence score categories^1^
