# Supplementary material for: Adherence to the World Cancer Research Fund/American Institute for Cancer Research and Korean Cancer Prevention Guidelines and cancer risk: a prospective cohort study from the Health Examinees-Gem study
Source: Epidemiol Health. 2023 Aug 1;45:e2023070. doi: 10.4178/epih.e2023070 (PMC10667577; doi:10.4178/epih.e2023070)
Supplement: Supplement Material 1. — Operationalization of the WCRF/AICR cancer prevention recommendations in the HEXA study. N (%) [file epih-45-e2023070-Supplementary-1.docx]

Supplementary Material 1. Operationalization of the WCRF/AICR cancer prevention recommendations in the HEXA study. N (%)

| **WCRF/AICR Recommendations** | **Operationalization of Recommendations** | **Points** | **Men** | **Women** |
| --- | --- | --- | --- | --- |
| Be a healthy weight: BMI (kg/m^2^), Waist circumference (cm) | 18.5-22.9 | 0.50 | 10262 (28.3) | 29561 (43.4) |
|  | 23.0-24.9 | 0.25 | 10966 (30.2) | 18088 (26.6) |
|  | <18.5 or ≥25 | 0.00 | 15038 (41.5) | 20481 (30.0) |
|  | M: <90, W: <80 | 0.5 | 25760 (71.0) | 54146 (79.5) |
|  | M: ≥90, W: ≥80 | 0.0 | 10506 (29.0) | 13984 (20.5) |
| Be physically active: Total moderate-vigorous physical activity (min/wk) | ≥150 | 1.0 | 15658 (43.2) | 25487 (37.4) |
|  | 75–<150 | 0.5 | 3365 (9.2) | 6199 (9.1) |
|  | <75 | 0.0 | 17243 (47.6) | 36444 (53.5) |
| Eat a better diet: Fruits and vegetables (g/day) | ≥400 | 1.0 | 5871 (16.2) | 9959 (14.6) |
|  | 200–<400 | 0.5 | 16220 (44.7) | 29557 (43.4) |
|  | <200 | 0.0 | 14175 (39.1) | 28614 (42.0) |
| Limit “fast foods”: Total ultra-processed foods (g/day) | Tertile 1:<16.0 | 1.0 | 12030 (33.2) | 22647 (33.2) |
|  | Tertile 2:16.0-42.2 | 0.5 | 12158 (33.5) | 22808 (33.5) |
|  | Tertile 3: ≥42.3 | 0.0 | 12078 (33.3) | 22675 (33.3) |
| Limit red and processed meat: Total red meat (g/wk) and processed meat (g/wk) | Red meat <500 and processed meat <21 | 1.0 | 25434 (70.1) | 54586 (80.1) |
|  | Red meat <500 and processed meat 21–<100 | 0.5 | 1975 (5.5) | 3643 (5.4) |
|  | Red meat >500 or processed meat ≥100 | 0.0 | 8857 (24.4) | 9901 (14.5) |
| Cut down on sugary drinks: Total sugar-sweetened drinks (g/day) | 0 | 1.0 | 521 (1.5) | 866 (1.2) |
|  | >0–≤250 | 0.5 | 34352 (94.7) | 64961 (95.4) |
|  | >250 | 0.0 | 1393 (3.8) | 2303 (3.4) |
| Limit alcohol consumption: Total ethanol (g/day) | 0 | 1.0 | 10036 (27.7) | 47451 (69.7) |
|  | >0–≤28 (2 drinks) males and ≤14 (1 drink) females | 0.5 | 19788 (54.6) | 18455 (27.0) |
|  | >28 (2 drinks) males and >14 (1 drink) females | 0.0 | 6442 (17.7) | 2224 (3.3) |
| Breastfeed your baby, if you can: Cumulative duration of breastfeeding throughout lifetime (month) | 6+ months | 1.0 |  | 49471 (72.6) |
|  | >0–<6 months | 0.5 |  | 5960 (8.8) |
|  | Never | 0.0 |  | 12699 (18.6) |
| Do not use supplements for cancer prevention | Not included |  |  |  |
| After a cancer diagnosis | Not included |  |  |  |
