# Supplementary material for: Adherence to the World Cancer Research Fund/American Institute for Cancer Research and Korean Cancer Prevention Guidelines and cancer risk: a prospective cohort study from the Health Examinees-Gem study
Source: Epidemiol Health. 2023 Aug 1;45:e2023070. doi: 10.4178/epih.e2023070 (PMC10667577; doi:10.4178/epih.e2023070)
Supplement: Supplement Material 2. — Operationalization of the Korea cancer prevention recommendations in the HEXA study. N (%) [file epih-45-e2023070-Supplementary-2.docx]

Supplementary Material 2. Operationalization of the Korea cancer prevention recommendations in the HEXA study. N (%)

| **Korea cancer prevention recommendations** | **Operationalization of Recommendations** | **Points** | **Men** | **Women** |
| --- | --- | --- | --- | --- |
| Limit smoking: Smoking status | Never | 1.0 | 9791 (27.0) | 65688 (96.4) |
|  | Former | 0.5 | 14811 (40.8) | 858 (1.3) |
|  | Current | 0.0 | 11664 (32.2) | 1584 (2.3) |
| Eat plenty of vegetables and fruits: Fruits and vegetables (g/day) | ≥400 | 1.0 | 5871 (16.2) | 9959 (14.6) |
|  | 200–<400 | 0.5 | 16220 (44.7) | 29557 (43.4) |
|  | <200 | 0.0 | 14175 (39.1) | 28614 (42.0) |
| Eat food without salty: Total Sodium intake (g/mg) | <1500 | 1.0 | 7422 (20.5) | 17938 (26.3) |
|  | 1500–<2300 | 0.5 | 8784 (24.2) | 18236 (26.8) |
|  | ≥2300 | 0.0 | 20060 (55.3) | 31956 (46.9) |
| Limit alcohol consumption: Total ethanol (g/day) | 0 | 1.0 | 10036 (27.7) | 47451 (69.7) |
|  | >0–≤28 (2 drinks) men and ≤14 (1 drink) women | 0.5 | 19788 (54.6) | 18455 (27.1) |
|  | >28 (2 drinks) men and >14 (1 drink) women | 0.0 | 6442 (17.8) | 2224 (3.3) |
| 5 or more times a week, 30 minutes or more a day, walking or exercising enough to sweat: Total moderate-vigorous physical activity (min/wk) | ≥150 | 1.0 | 15658 (43.2) | 25487 (37.4) |
|  | 75–<150 | 0.5 | 3365 (9.3) | 6199 (9.1) |
|  | <75 | 0.0 | 17243 (47.6) | 36444 (53.5) |
| Maintain a healthy weight: BMI(kg/m2, Waist circumference (cm)) | 18.5-22.9 | 0.50 | 10262 (28.3) | 29561 (43.4) |
|  | 23.0-24.9 | 0.25 | 10966 (30.2) | 18088 (26.6) |
|  | <18.5 or ≥25 | 0.00 | 15038 (41.5) | 20481 (30.1) |
|  | Men: <90, Women: <80 | 0.5 | 25760 (71.0) | 54146 (79.5) |
|  | Men: ≥90, Women: ≥80 | 0.0 | 10506 (29.0) | 13984 (20.5) |
| Hepatitis B and cervical cancer vaccination | Not included |  |  |  |
| Have a safe sexual health | Not included |  |  |  |
| Observe safety and health rules | Not included |  |  |  |
| Have a cancer screening | Not included |  |  |  |
