# Supplementary material for: Adherence to the World Cancer Research Fund/American Institute for Cancer Research and Korean Cancer Prevention Guidelines and cancer risk: a prospective cohort study from the Health Examinees-Gem study
Source: Epidemiol Health. 2023 Aug 1;45:e2023070. doi: 10.4178/epih.e2023070 (PMC10667577; doi:10.4178/epih.e2023070)
Supplement: Supplement Material 3. — Baseline characteristics of the participants according to Korean cancer prevention guideline categories and gender. [file epih-45-e2023070-Supplementary-3.docx]

Supplementary Material 3. Baseline characteristics of the participants according to Korean cancer prevention guideline categories and gender.

|  | WCRF/AICR Adherence Score | | | | | | |  |
| --- | --- | --- | --- | --- | --- | --- | --- | --- |
|  |  | Men (n=36,266) |  |  |  | Women (n=68,130) |  |  |
|  | Tertile1 | Tertile2 | Tertile3 | *p*-value^1^ | Tertile1 | Tertile2 | Tertile3 | *p*-value^1^ |
| Score range | 0≤score<2.50 | 2.50≤score<3.25 | score≥3.25 |  | 0≤score<3.50 | 3.50≤score<4.00 | score≥4.00 |  |
| No. of participants | 11402 (31.4) | 12139 (33.5) | 12725 (35.1) | <.001 | 21979 (32.3) | 17065 (25.1) | 29086 (42.7) | <.001 |
| Age (yr) | 51.6±8.2 | 53.6±8.3 | 55.2±8.2 | <.001 | 52.2±8 | 52.0±7.8 | 52.6±7.5 | <.001 |
| Body mass index (kg/m^2^) | 25.3±2.8 | 24.5±2.9 | 23.5±2.2 | <.001 | 25.1±3.2 | 23.4±2.8 | 22.6±2.4 | <.001 |
| Education |  |  |  | <.001 |  |  |  | <.001 |
| ≤Middle school | 2565 (22.5) | 2513 (20.7) | 2248 (17.7) |  | 9103 (41.4) | 5921 (34.7) | 9101 (31.3) |  |
| High school | 4926 (43.2) | 4947 (40.8) | 4935 (38.8) |  | 8897 (40.5) | 7346 (43.1) | 13344 (45.9) |  |
| ≥College | 3801 (33.3) | 4573 (37.7) | 5439 (42.7) |  | 3823 (17.4) | 3668 (21.4) | 6459 (22.2) |  |
| missing | 110 (1.0) | 106 (0.8) | 103 (0.8) |  | 156 (0.7) | 130 (0.8) | 182 (0.6) |  |
| Income (10^4^ Korean won) |  |  |  | <.001 |  |  |  | <.001 |
| <200 | 2678 (23.5) | 2988 (24.5) | 2948 (23.1) |  | 7446 (33.9) | 5006 (29.3) | 7812 (26.9) |  |
| 200-400 | 5088 (44.6) | 5157 (42.5) | 5466 (43.0) |  | 8477 (38.6) | 6766 (39.7) | 11586 (39.8) |  |
| ≥400 | 3013 (26.4) | 3344 (27.6) | 3621 (28.5) |  | 4482 (20.4) | 4115 (24.1) | 7705 (26.5) |  |
| missing | 623 (5.5) | 650 (5.4) | 690 (5.4) |  | 1574 (7.1) | 1178 (6.9) | 1983 (6.8) |  |
| Smoking status |  |  |  | <.001 |  |  |  | <.001 |
| Never | 615 (5.4) | 2728 (22.5) | 6448 (50.7) |  | 19995 (91.0) | 16779 (98.3) | 28914 (99.4) |  |
| Former | 3663 (32.1) | 5829 (48.0) | 5319 (41.8) |  | 562 (2.5) | 169 (1.0) | 127 (0.4) |  |
| Current | 7124 (62.5) | 3582 (29.5) | 958 (7.5) |  | 1422 (6.5) | 117 (0.7) | 45 (0.2) |  |
| Missing |  |  |  |  |  |  |  |  |
| Alcohol intake, (g of ethanol/day) | 26.2±45.4 | 14.3±24.9 | 6.8±16.3 | <.001 | 3.9±14.1 | 1.7±30.5 | 0.7±3.2 | <.001 |
| Physical activity(min/wk) |  |  |  | <.001 |  |  |  | <.001 |
| <75 | 9287(81.5) | 5753(47.4) | 2203(17.3) |  | 19517(88.8) | 11317(66.3) | 5610(19.3) |  |
| 75-149 | 800 (7.0) | 1360 (11.2) | 1205 (9.5) |  | 1180 (5.4) | 1846 (10.8) | 3173 (10.9) |  |
| ≥150 | 1315 (11.5) | 5026 (41.4) | 9317 (73.2) |  | 1282 (5.8) | 3902 (22.9) | 20303 (69.8) |  |
| Family history of cancer |  |  |  | 0.226 |  |  |  | 0.027 |
| No | 8419 (73.8) | 8890 (73.2) | 9228 (72.5) |  | 15571 (70.8) | 12026 (70.5) | 20313 (69.8) |  |
| Yes | 2960 (26.0) | 3223 (26.6) | 3466 (27.2) |  | 6346 (28.9) | 5002 (29.3) | 8715 (30.0) |  |
| Missing | 23 (0.2) | 26 (0.2) | 31 (0.2) |  | 62 (0.3) | 37 (0.2) | 58 (0.2) |  |
| Breastfeeding(mo) |  |  |  |  |  |  |  | 0.002 |
| None |  |  |  |  | 4105 (18.7) | 3219 (18.9) | 5375 (18.5) |  |
| <6 |  |  |  |  | 1812 (8.2) | 1595 (9.3) | 2553 (8.8) |  |
| ≥6 |  |  |  |  | 16062 (73.1) | 12251 (71.8) | 21158 (72.7) |  |
| Energy intake (kcal/day) | 1883.3±497.2 | 1842.5±492.2 | 1813±497.1 | <.001 | 1716.4±496 | 1691.7±504 | 1667.2±513 | <.001 |
| Vegetable and fruits intake (g/day) | 261.2±152.4 | 268.8±179.7 | 275.5±201.7 | <.001 | 252.1±149.8 | 257.5±173.5 | 267.6±198.7 | <.001 |
| Fast food intake (g/day) | 56.7±162.1 | 182.1±259.4 | 309.2±287 | <.001 | 40.1±51.7 | 36.2±44 | 31.8±41.6 | <.001 |
| Red meat intake (g/day) | 59.7±52.4 | 52.8±49.3 | 47.2±48.7 | <.001 | 41.8±45.5 | 38.5±43.1 | 35.2±40.9 | <.001 |
| Sugar-sweetened drinks (g/day) | 59.1±88.6 | 58.2±82 | 57.8±79.7 | <.001 | 55.2±78 | 56.3±78.2 | 57.5±78.7 | <.001 |
| Sodium intake (g/day) | 2763.8±1245.9 | 2656±1416.1 | 2532.1±1558.3 | <.001 | 2516.1±1154.3 | 2412.4±1323.4 | 2282.6±1438 | <.001 |

The distribution of variables was reported as n (%) and Means ± SD.

^1^*p* values were conducted by Chi-square test (categorical variables) and generalized linear model (continuous variables)
