# Supplementary material for: Adherence to the World Cancer Research Fund/American Institute for Cancer Research and Korean Cancer Prevention Guidelines and cancer risk: a prospective cohort study from the Health Examinees-Gem study
Source: Epidemiol Health. 2023 Aug 1;45:e2023070. doi: 10.4178/epih.e2023070 (PMC10667577; doi:10.4178/epih.e2023070)
Supplement: Supplement Material 4. — Associations between adherence to individual components of the 2 cancer prevention guideline score and cancer risk in men. [file epih-45-e2023070-Supplementary-4.docx]

Supplementary Material 4. Associations between adherence to individual components of the 2 cancer prevention guideline score and cancer risk in men.

|  | Stomach cancer | | | Colorectal cancer | | | Lung cancer | | | Prostate cancer | | |
| --- | --- | --- | --- | --- | --- | --- | --- | --- | --- | --- | --- | --- |
| Components of the cancer prevention guideline score | No.of cases/total participants | Crude HR (95%CI) | Multivariable adjusted HR (95%CI) | No.of cases/total participants | Crude HR (95%CI) | Multivariable adjusted HR (95%CI) | No.of cases/total participants | Crude HR (95%CI) | Multivariable adjusted HR (95%CI) | No.of cases/total participants | Crude HR (95%CI) | Multivariable adjusted HR (95%CI) |
| Be a healthy weight (BMI) ^1,3,4^ | |  |  |  |  |  |  |  |  |  |  |  |
| 0.00 | 222/15038 | 1.00 | 1.00 | 184/15038 | 1.00 | 1.00 | 101/15038 | 1.00 | 1.00 | 197/15038 | 1.00 | 1.00 |
| 0.25 | 135/10966 | 0.80  (0.65-0.99) | 0.80  (0.64-0.99) | 114/10966 | 0.82  (0.65-1.03) | 0.81  (0.64-1.03) | 91/10966 | 1.15  (0.87-1.53) | 1.15  (0.87-1.53) | 143/10966 | 0.91  (0.74-1.13) | 0.73  (0.58-0.93) |
| 0.50 | 141/10262 | 0.90  (0.73-1.11) | 0.89  (0.72-1.1) | 99/10262 | 0.77  (0.60-0.98) | 0.76  (0.60-0.97) | 99/10262 | 1.32  (1.00-1.75) | 1.25  (0.95-1.66) | 109/10262 | 0.92  (0.74-1.14) | 0.75  (0.59-0.94) |
| Be a healthy weight (Waist circumference)) ^1,3,4^ | | | |  |  |  |  |  |  |  |  |  |
| 0.0 | 166/10506 | 1.00 | 1.00 | 147/10506 | 1.00 | 1.00 | 88/10506 | 1.00 | 1.00 | 156/10506 | 1.00 | 1.00 |
| 0.5 | 332/25760 | 0.87  (0.72-1.05) | 0.87  (0.72-1.05) | 250/25760 | 0.74  (0.60-0.90) | 0.73  (0.60-0.90) | 203/25760 | 1.03  (0.80-1.32) | 1.05  (0.82-1.35) | 293/25760 | 0.85  (0.7-1.04) | 0.85  (0.7-1.04) |
| Be physically active^1,3,4^ | |  |  |  |  |  |  |  |  |  |  |  |
| 0.0 | 238/17243 | 1.00 | 1.00 | 182/17243 | 1.00 | 1.00 | 160/17243 | 1.00 | 1.00 | 198/17243 | 1.00 | 1.00 |
| 0.5 | 34/3365 | 0.77  (0.54-1.11) | 0.81  (0.56-1.16) | 41/3365 | 1.20  (0.86-1.69) | 1.22  (0.86-1.71) | 21/3365 | 0.76  (0.48-1.19) | 0.91  (0.58-1.44) | 32/3365 | 0.96  (0.66-1.39) | 1.01  (0.84-1.23) |
| 1.0 | 226/15658 | 0.95  (0.79-1.15) | 1.00  (0.82-1.20) | 174/15658 | 0.96  (0.78-1.19) | 0.97  (0.79-1.21) | 110/15658 | 0.65  (0.51-0.82) | 0.79  (0.62-1.02) | 219/15658 | 0.88  (0.60-1.28) | 0.92  (0.76-1.12) |
| Eat a better diet^1,3,4^ | | | | | |  |  |  |  |  |  |  |
| 0.0 | 207/14175 | 1.00 | 1.00 | 152/14175 | 1.00 | 1.00 | 115/14175 | 1.00 | 1.00 | 162/14175 | 1.00 | 1.00 |
| 0.5 | 224/16220 | 0.96  (0.79-1.16) | 0.94  (0.77-1.15) | 183/16220 | 1.07  (0.86-1.32) | 1.10  (0.88-1.37) | 131/16220 | 1.02  (0.79-1.31) | 1.00  (0.78-1.29) | 213/16220 | 1.18  (0.96-1.45) | 1.14  (0.87-1.50) |
| 1.0 | 67/5871 | 0.79  (0.60-1.04) | 0.75  (0.56-1.00) | 62/5871 | 0.99  (0.74-1.33) | 1.06  (0.77-1.46) | 45/5871 | 0.97  (0.69-1.37) | 0.97  (0.68-1.40) | 74/5871 | 1.16  (0.94-1.44) | 1.09  (0.81-1.47) |
| Limit “fast foods” ^1,3^ | | | | | | | |  |  |  |  |  |
| 0.0 | 127/12078 | 1.00 | 1.00 | 117/12078 | 1.00 | 1.00 | 64/12078 | 1.00 | 1.00 | 120/12078 | 1.00 | 1.00 |
| 0.5 | 168/12158 | 1.15  (0.91-1.45) | 1.20  (0.94-1.52) | 123/12158 | 0.91  (0.70-1.17) | 0.86  (0.66-1.13) | 101/12158 | 1.26  (0.92-1.72) | 1.30  (0.94-1.80) | 149/12158 | 0.94  (0.74-1.20) | 0.80  (0.63-1.00) |
| 1.0 | 203/12030 | 1.16  (0.92-1.45) | 1.22  (0.96-1.56) | 157/12030 | 0.98  (0.77-1.25) | 0.91  (0.69-1.19) | 126/12030 | 1.15  (0.85-1.56) | 1.20  (0.87-1.65) | 180/12030 | 0.97  (0.76-1.24) | 0.83  (0.65-1.06) |
| Limit red and processed meat ^1,3^ | | | |  |  |  |  |  |  |  |  |  |
| 0.0 | 103/8857 | 1.00 | 1.00 | 87/8857 | 1.00 | 1.00 | 65/8857 | 1.00 | 1.00 | 92/8857 | 1.00 | 1.00 |
| 0.5 | 19/1975 | 1.05  (0.64-1.72) | 1.08  (0.66-1.77) | 10/1975 | 0.65  (0.34-1.26) | 0.64  (0.33-1.24) | 10/1975 | 1.03  (0.53-2.00) | 1.03  (0.53-2.02) | 7/1975 | 0.56  (0.26-1.19) | 0.97  (0.77-1.22) |
| 1.0 | 376/25434 | 1.09  (0.87-1.35) | 1.15  (0.90-1.47) | 300/25434 | 1.04  (0.82-1.31) | 0.98  (0.76-1.27) | 216/25434 | 0.89  (0.67-1.17) | 0.93  (0.68-1.27) | 350/25434 | 0.57  (0.26-1.23) | 1.02  (0.79-1.31) |
| Cut down on sugary drinks^1,3^ | | | |  |  |  |  |  |  |  |  |  |
| 0.0 | 18/1393 | 1.00 | 1.00 | 16/1393 | 1.00 | 1.00 | 10/1393 | 1.00 | 1.00 | 14/1393 | 1.00 | 1.00 |
| 0.5 | 466/34352 | 0.91  (0.57-1.46) | 0.95  (0.58-1.54) | 372/34352 | 0.82  (0.50-1.36) | 0.76  (0.46-1.27) | 277/34352 | 0.88  (0.47-1.66) | 0.84  (0.44-1.62) | 427/34352 | 0.93  (0.54-1.58) | 0.92  (0.38-2.19) |
| 1.0 | 14/521 | 1.62  (0.80-3.27) | 1.69  (0.83-3.46) | 9/521 | 1.18  (0.52-2.69) | 1.05  (0.46-2.40) | 4/521 | 0.70  (0.22-2.22) | 0.61  (0.19-1.99) | 8/521 | 1.01  (0.59-1.75) | 1.10  (0.45-2.67) |
| Limit alcohol consumption^1,3,4^ | | |  |  |  |  |  |  |  |  |  |  |
| 0.0 | 89/6442 | 1.00 | 1.00 | 72/6442 | 1.00 | 1.00 | 53/6442 | 1.00 | 1.00 | 53/6442 | 1.00 | 1.00 |
| 0.5 | 272/19788 | 0.91  (0.72-1.16) | 0.95  (0.75-1.21) | 208/19788 | 0.88  (0.67-1.15) | 0.88  (0.67-1.15) | 141/19788 | 0.73  (0.53-1.00) | 0.85  (0.62-1.18) | 226/19788 | 1.14  (0.84-1.54) | 1.31  (0.96-1.78) |
| 1.0 | 137/10036 | 0.80  (0.61-1.04) | 0.86  (0.66-1.13) | 117/10036 | 0.87  (0.64-1.16) | 0.87  (0.64-1.17) | 97/10036 | 0.79  (0.57-1.11) | 1.08  (0.77-1.52) | 170/10036 | 1.09  (0.80-1.47) | 1.22  (0.89-1.68) |
| Limit smoking^2,4^ |  |  |  |  |  |  |  |  |  |  |  |  |
| 0.0 | 174/11664 | 1.00 | 1.00 | 108/11664 | 1.00 | 1.00 | 147/11664 | 1.00 | 1.00 | 75/11664 | 1.00 | 1.00 |
| 0.5 | 204/14811 | 0.72  (0.58-0.88) | 0.71  (0.58-0.88) | 180/14811 | 1.06  (0.83-1.35) | 1.06  (0.83-1.35) | 108/14811 | 0.37  (0.29-0.48) | 0.38  (0.29-0.49) | 218/14811 | 1.42  (1.09-1.84) | 1.54  (1.17-2.02) |
| 1.0 | 120/9791 | 0.64  (0.51-0.81) | 0.64  (0.51-0.81) | 109/9791 | 0.98  (0.75-1.28) | 0.99  (0.75-1.3) | 36/9791 | 0.19  (0.13-0.27) | 0.19  (0.13-0.28) | 156/9791 | 1.39  (1.07-1.81) | 1.50  (1.14-1.98) |
| Eat food without salty^1,4^ | |  |  |  |  |  |  |  |  |  | 1.00 | 1.00 |
| 0.0 | 265/20060 | 1.00 | 1.00 | 218/20060 | 1.00 | 1.00 | 156/20060 | 1.00 | 1.00 | 241/20060 |  |  |
| 0.5 | 129/8784 | 1.09  (0.89-1.35) | 1.12  (0.90-1.39) | 94/8784 | 0.97  (0.76-1.24) | 0.94  (0.73-1.2) | 70/8784 | 0.99  (0.75-1.31) | 1.02  (0.77-1.36) | 111/8784 | 1.00  (0.80-1.26) | 0.96  (0.76-1.21) |
| 1.0 | 104/7422 | 0.99  (0.79-1.24) | 1.02  (0.80-1.30) | 85/7422 | 0.99  (0.77-1.27) | 0.92  (0.71-1.21) | 65/7422 | 1.01  (0.76-1.35) | 1.07  (0.79-1.46) | 97/7422 | 1.03  (0.82-1.29) | 1.00  (0.77-1.29) |

^1^Adjusted for education level (less than high school, high school, college or above and missing), smoking status (nonsmoker, ex-smoker, and current smoker, missing), total energy intake (tertiles), and family history of cancer (yes, no, missing).

^2^Adjusted for level (less than high school, high school, college or above and missing), total energy intake (tertiles), and family history of cancer (yes, no, missing).

^3^ Components of the WCRF/AICR score.

^4^ Components of the Korean Cancer Prevention Guidelines score.
