# Supplementary material for: Adherence to the World Cancer Research Fund/American Institute for Cancer Research and Korean Cancer Prevention Guidelines and cancer risk: a prospective cohort study from the Health Examinees-Gem study
Source: Epidemiol Health. 2023 Aug 1;45:e2023070. doi: 10.4178/epih.e2023070 (PMC10667577; doi:10.4178/epih.e2023070)
Supplement: Supplement Material 5. — Associations between adherence to individual components of the 2 cancer prevention guideline score and cancer risk in women. [file epih-45-e2023070-Supplementary-5.docx]

Supplementary Material 5. Associations between adherence to individual components of the 2 cancer prevention guideline score and cancer risk in women.

|  | Stomach cancer | | | Colorectal cancer | | | Lung cancer | | | Breast cancer | | |
| --- | --- | --- | --- | --- | --- | --- | --- | --- | --- | --- | --- | --- |
| Components of the cancer prevention guideline score | No.of cases/total participants | Crude HR (95%CI) | Multivariable adjusted HR (95%CI) | No.of cases/total participants | Crude HR (95%CI) | Multivariable adjusted HR (95%CI) | No.of cases/total participants | Crude HR (95%CI) | Multivariable adjusted HR (95%CI) | No.of cases/total participants | Crude HR (95%CI) | Multivariable adjusted HR (95%CI) |
| Be a healthy weight (BMI) ^1,3,4^ | |  |  |  |  |  |  |  |  |  |  |  |
| 0.00 | 138/20481 | 1.00 | 1.00 | 132/20481 | 1.00 | 1.00 | 68/20481 | 1.00 | 1.00 | 265/20481 | 1.00 | 1.00 |
| 0.25 | 102/18088 | 0.87  (0.67-1.12) | 0.87  (0.67-1.12) | 103/18088 | 0.91  (0.70-1.18) | 0.90  (0.70-1.17) | 65/18088 | 1.17  (0.83-1.65) | 1.16  (0.82-1.62) | 210/18088 | 0.88  (0.73-1.05) | 0.86  (0.72-1.03) |
| 0.50 | 162/29561 | 0.96  (0.76-1.21) | 0.95  (0.76-1.20) | 153/29561 | 0.96  (0.76-1.22) | 0.94  (0.74-1.19) | 110/29561 | 1.53  (1.13-2.08) | 1.48  (1.09-2.00) | 387/29561 | 0.96  (0.82-1.13) | 0.90  (0.77-1.06) |
| Be a healthy weight (Waist circumference)) ^1,3,4^ | | | |  |  |  |  |  |  |  |  |  |
| 0.0 | 103/13984 | 1.00 | 1.00 | 97/13984 | 1.00 | 1.00 | 67/13984 | 1.00 | 1.00 | 171/13984 | 1.00 | 1.00 |
| 0.5 | 299/54146 | 0.92  (0.73-1.15) | 0.91  (0.72-1.14) | 291/54146 | 0.96  (0.76-1.20) | 0.94  (0.75-1.18) | 176/54146 | 0.96  (0.72-1.28) | 0.92  (0.69-1.23) | 691/54146 | 1.00  (0.84-1.18) | 0.93  (0.79-1.11) |
| Be physically active^1,3,4^ | |  |  |  |  |  |  |  |  |  |  |  |
| 0.0 | 198/36444 | 1.00 | 1.00 | 203/36444 | 1.00 | 1.00 | 129/36444 | 1.00 | 1.00 | 470/36444 | 1.00 | 1.00 |
| 0.5 | 42/6199 | 1.25  (0.90-1.74) | 1.24  (0.89-1.73) | 37/6199 | 1.06  (0.75-1.51) | 1.05  (0.74-1.49) | 18/6199 | 0.83  (0.51-1.36) | 0.81  (0.49-1.32) | 84/6199 | 1.04  (0.83-1.32) | 1.00  (0.80-1.27) |
| 1.0 | 162/25487 | 1.16  (0.94-1.43) | 1.15  (0.93-1.42) | 148/25487 | 1.01  (0.82-1.25) | 1.01  (0.81-1.25) | 96/25487 | 1.04  (0.80-1.36) | 1.02  (0.78-1.33) | 308/25487 | 0.97  (0.84-1.12) | 0.94  (0.82-1.09) |
| Eat a better diet^1,3,4^ | | | | | |  |  |  |  |  |  |  |
| 0.0 | 160/28614 | 1.00 | 1.00 | 160/28614 | 1.00 | 1.00 | 102/28614 | 1.00 | 1.00 | 372/28614 |  | 1.00 |
| 0.5 | 176/29557 | 1.06  (0.86-1.32) | 1.08  (0.87-1.36) | 179/29557 | 1.08  (0.87-1.33) | 1.07  (0.85-1.33) | 111/29557 | 1.06  (0.81-1.38) | 1.00  (0.75-1.32) | 371/29557 | 0.96  (0.83-1.11) | 0.97  (0.83-1.12) |
| 1.0 | 66/9959 | 1.13  (0.85-1.50) | 1.19  (0.88-1.60) | 49/9959 | 0.83  (0.60-1.15) | 0.81  (0.58-1.14) | 30/9959 | 0.81  (0.54-1.21) | 0.73  (0.47-1.13) | 119/9959 | 0.88  (0.72-1.08) | 0.89  (0.72-1.11) |
| Limit “fast foods” ^1,3^ | | | | | | | |  |  |  |  |  |
| 0.0 | 98/22675 | 1.00 | 1.00 | 111/22675 | 1.00 | 1.00 | 80/22675 | 1.00 | 1.00 | 324/22675 | 1.00 | 1.00 |
| 0.5 | 125/22808 | 1.12  (0.86-1.47) | 1.13  (0.85-1.49) | 126/22808 | 0.95  (0.74-1.23) | 0.97  (0.75-1.26) | 71/22808 | 0.68  (0.49-0.94) | 0.70  (0.51-0.97) | 280/22808 | 0.88  (0.75-1.04) | 0.89  (0.75-1.04) |
| 1.0 | 179/22647 | 1.37  (1.07-1.77) | 1.40  (1.07-1.84) | 151/22647 | 0.95  (0.74-1.22) | 0.98  (0.75-1.28) | 92/22647 | 0.65  (0.48-0.89) | 0.70  (0.50-0.97) | 258/22647 | 0.85  (0.71-1.01) | 0.88  (0.73-1.05) |
| Limit red and processed meat ^1,3^ | | | |  |  |  |  |  |  |  |  |  |
| 0.0 | 54/9901 | 1.00 | 1.00 | 47/9901 | 1.00 | 1.00 | 40/9901 | 1.00 | 1.00 | 128/9901 | 1.00 | 1.00 |
| 0.5 | 12/3643 | 0.75  (0.4-1.42) | 0.73  (0.39-1.39) | 18/3643 | 1.42  (0.82-2.45) | 1.41  (0.81-2.43) | 4/3643 | 0.41  (0.14-1.14) | 0.39  (0.14-1.11) | 48/3643 | 1.01  (0.72-1.40) | 0.96  (0.68-1.34) |
| 1.0 | 336/54586 | 0.98  (0.73-1.3) | 0.94  (0.70-1.26) | 323/54586 | 1.04  (0.77-1.42) | 1.06  (0.77-1.46) | 199/54586 | 0.70  (0.50-0.99) | 0.73  (0.50-1.06) | 686/54586 | 1.00  (0.83-1.21) | 0.98  (0.81-1.20) |
| Cut down on sugary drinks^1,3^ | | | |  |  |  |  |  |  |  |  |  |
| 0.0 | 10/2303 | 1.00 | 1.00 | 3/2303 | 1.00 | 1.00 | 11/2303 | 1.00 | 1.00 | 32/2303 | 1.00 | 1.00 |
| 0.5 | 385/64961 | 1.34  (0.71-2.50) | 1.30  (0.69-2.45) | 381/64961 | 4.41  (1.41-13.74) | 4.65  (1.49-14.57) | 231/64961 | 0.69  (0.38-1.26) | 0.73  (0.39-1.35) | 818/64961 | 0.96  (0.67-1.36) | 0.96  (0.67-1.37) |
| 1.0 | 7/866 | 1.61  (0.61-4.23) | 1.58  (0.59-4.22) | 4/866 | 3.07  (0.69-13.75) | 3.39  (0.76-15.25) | 1/866 | 0.18  (0.02-1.42) | 0.20  (0.03-1.59) | 12/866 | 1.08  (0.56-2.10) | 1.15  (0.59-2.25) |
| Limit alcohol consumption^1,3,4^ | | |  |  |  |  |  |  |  |  |  |  |
| 0.0 | 8/2224 | 1.00 | 1.00 | 13/2224 | 1.00 | 1.00 | 7/2224 | 1.00 | 1.00 | 33/2224 | 1.00 | 1.00 |
| 0.5 | 96/18455 | 1.34  (0.65-2.75) | 1.25  (0.6-2.62) | 89/18455 | 0.76  (0.43-1.36) | 0.75  (0.41-1.37) | 50/18455 | 0.74  (0.34-1.64) | 0.81  (0.36-1.79) | 239/18455 | 0.87  (0.61-1.26) | 0.83  (0.57-1.19) |
| 1.0 | 298/47451 | 1.31  (0.64-2.65) | 1.21  (0.58-2.5) | 286/47451 | 0.75  (0.43-1.31) | 0.73  (0.40-1.31) | 186/47451 | 0.73  (0.34-1.57) | 0.80  (0.37-1.75) | 590/47451 | 0.87  (0.61-1.24) | 0.81  (0.57-1.16) |
| For mothers: breastfeed your baby^1,3^ | | |  |  |  |  |  |  |  |  |  |  |
| 0.0 | 46/12699 | 1.00 | 1.00 | 60/12699 | 1.00 | 1.00 | 29/12699 | 1.00 | 1.00 | 209/12699 | 1.00 | 1.00 |
| 0.5 | 28/5960 | 1.39  (0.87-2.22) | 1.38  (0.86-2.21) | 25/5960 | 0.98  (0.62-1.56) | 0.97  (0.61-1.55) | 15/5960 | 1.32  (0.71-2.47) | 1.30  (0.70-2.43) | 80/5960 | 0.79  (0.61-1.02) | 0.78  (0.60-1.01) |
| 1.0 | 328/49471 | 1.41  (1.02-1.95) | 1.41  (1.02-1.96) | 303/49471 | 0.93  (0.70-1.24) | 0.98  (0.73-1.31) | 199/49471 | 1.09  (0.73-1.61) | 1.26  (0.84-1.87) | 573/49471 | 0.73  (0.62-0.85) | 0.79  (0.67-0.94) |
| Limit smoking^2,4^ |  |  |  |  |  |  |  |  |  |  |  |  |
| 0.0 | 5/1584 | 1.00 | 1.00 | 8/1584 | 1.00 | 1.00 | 8/1584 | 1.00 | 1.00 | 16/1584 | 1.00 | 1.00 |
| 0.5 | 2/858 | 0.69  (0.13-3.55) | 0.69  (0.13-3.57) | 4/858 | 0.88  (0.26-2.91) | 0.86  (0.26-2.84) | 4/858 | 0.81  (0.25-2.68) | 0.78  (0.24-2.58) | 13/858 | 1.49  (0.72-3.10) | 1.42  (0.68-2.96) |
| 1.0 | 395/65688 | 1.60  (0.66-3.88) | 1.62  (0.67-3.91) | 376/65688 | 0.94  (0.47-1.89) | 0.92  (0.46-1.86) | 231/65688 | 0.52  (0.25-1.05) | 0.49  (0.24-0.99) | 833/65688 | 1.26  (0.77-2.07) | 1.20  (0.73-1.97) |
| Eat food without salty^1,4^ | |  |  |  |  |  |  |  |  |  | 1.00 | 1.00 |
| 0.0 | 195/31956 | 1.00 | 1.00 | 194/31956 | 1.00 | 1.00 | 111/31956 | 1.00 | 1.00 | 408/31956 | 1.00 | 1.00 |
| 0.5 | 103/18236 | 0.95  (0.75-1.21) | 0.93  (0.73-1.18) | 91/18236 | 0.84  (0.66-1.08) | 0.84  (0.65-1.08) | 60/18236 | 0.97  (0.71-1.33) | 1.02  (0.74-1.41) | 236/18236 | 1.04  (0.89-1.23) | 1.03  (0.87-1.21) |
| 1.0 | 104/17938 | 0.93  (0.74-1.18) | 0.90  (0.69-1.17) | 103/17938 | 0.93  (0.73-1.18) | 0.92  (0.71-1.19) | 72/17938 | 1.11  (0.83-1.49) | 1.24  (0.89-1.73) | 218/17938 | 0.98  (0.83-1.15) | 0.96  (0.80-1.15) |

^1^Adjusted for education level (less than high school, high school, college or above and missing), smoking status (nonsmoker, ex-smoker, and current smoker, missing), total energy intake (tertiles), and family history of cancer (yes, no, missing).

^2^Adjusted for level (less than high school, high school, college or above and missing), total energy intake (tertiles), and family history of cancer (yes, no, missing).

^3^ Components of the WCRF/AICR score.

^4^ Components of the Korean Cancer Prevention Guidelines score.
