# Supplementary material for: Adherence to the World Cancer Research Fund/American Institute for Cancer Research and Korean Cancer Prevention Guidelines and cancer risk: a prospective cohort study from the Health Examinees-Gem study
Source: Epidemiol Health. 2023 Aug 1;45:e2023070. doi: 10.4178/epih.e2023070 (PMC10667577; doi:10.4178/epih.e2023070)
Supplement: Supplement Material 6. — Hazard ratios (HRs) and 95% confidence intervals (CI) for sensitivity analysis of cancer risk according to WCRF/AICR cancer prevention guideline adherence score categories1 [file epih-45-e2023070-Supplementary-6.docx]

Supplementary Material 6. Hazard ratios (HRs) and 95% confidence intervals (CI) for sensitivity analysis of cancer risk according to WCRF/AICR cancer prevention guideline adherence score categories^1^

|  | Men (n=35,659) | | | | |  | Women (n=67,112) | | | | |  |
| --- | --- | --- | --- | --- | --- | --- | --- | --- | --- | --- | --- | --- |
|  | Tertile1 | Tertile2 | Tertile3 | *p* for trend^2^ | Continuous  (per 1-point increase in score) |  | Tertile1 | Tertile2 | Tertile3 | *p* for trend^2^ | Continuous  (per 1-point increase in score) | |
| Score range | 0≤score<3.25 | 3.25≤score<4.25 | score≥4.25 |  |  |  | 0≤score<4.50 | 4.50≤score<5.50 | score≥5.50 |  |  | |
| Person year | 96358.2 | 115217.2 | 107174.9 |  |  |  | 172592.7 | 214178 | 214995.1 |  |  | |
| Total cancer |  |  |  |  |  |  |  |  |  |  |  | |
| No.of cases/total subjects | 605/10802 | 812/12864 | 834/11993 |  |  |  | 905/19442 | 1173/23835 | 1193/23835 |  |  | |
| Crude HR (95%CI) | 1.00 | 0.92  (0.83-1.03) | 0.86  (0.77-0.95) | 0.005 | 0.95  (0.91-0.99) |  | 1.00 | 0.98  (0.9-1.07) | 0.96  (0.88-1.05) | 0.380 | 0.99  (0.96-1.02) | |
| Multivariable adjusted HR (95%CI) | 1.00 | 0.93  (0.83-1.03) | 0.87  (0.78-0.97) | 0.014 | 0.96  (0.92-1.00) |  | 1.00 | 0.99  (0.9-1.08) | 0.97  (0.89-1.07) | 0.546 | 0.99  (0.96-1.03) | |
| Stomach cancer |  |  |  |  |  |  |  |  |  |  |  | |
| No.of cases/total subjects | 109/10802 | 140/12864 | 144/11993 |  |  |  | 71/19442 | 110/23835 | 146/23835 |  |  | |
| Crude HR (95%CI) | 1.00 | 0.91  (0.71-1.18) | 0.87  (0.67-1.13) | 0.305 | 0.95  (0.86-1.05) |  | 1.00 | 1.08  (0.79-1.46) | 1.31  (0.97-1.76) | 0.062 | 1.17  (1.05-1.31) | |
| Multivariable adjusted HR (95%CI) | 1.00 | 0.92  (0.71-1.19) | 0.90  (0.69-1.19) | 0.464 | 0.97  (0.87-1.08) |  | 1.00 | 1.05  (0.78-1.43) | 1.27  (0.94-1.72) | 0.097 | 1.16  (1.04-1.29) | |
| Colorectal cancer |  |  |  |  |  |  |  |  |  |  |  | |
| No.of cases/total subjects | 82/10802 | 104/12864 | 103/11993 |  |  |  | 70/19442 | 118/23835 | 104/23835 |  |  | |
| Crude HR (95%CI) | 1.00 | 0.91(0.68-1.22) | 0.85  (0.63-1.15) | 0.294 | 0.93  (0.83-1.05) |  | 1.00 | 1.12  (0.83-1.52) | 0.89  (0.65-1.22) | 0.386 | 0.92  (0.82-1.02) | |
| Multivariable adjusted HR (95%CI) | 1.00 | 0.86(0.64-1.16) | 0.78  (0.57-1.06) | 0.109 | 0.90  (0.79-1.02) |  | 1.00 | 1.15  (0.85-1.56) | 0.92  (0.67-1.26) | 0.510 | 0.93  (0.83-1.04) | |
| Lung cancer |  |  |  |  |  |  |  |  |  |  |  | |
| No.of cases/total subjects | 67/10802 | 91/12864 | 84/11993 |  |  |  | 49/19442 | 80/23835 | 79/23835 |  |  | |
| Crude HR (95%CI) | 1.00 | 0.85  (0.62-1.17) | 0.66  (0.48-0.91) | 0.009 | 0.87  (0.78-0.98) |  | 1.00 | 0.97  (0.68-1.39) | 0.83  (0.58-1.19) | 0.273 | 0.96  (0.84-1.09) | |
| Multivariable adjusted HR (95%CI) | 1.00 | 0.92  (0.67-1.27) | 0.82  (0.59-1.15) | 0.249 | 0.97  (0.85-1.10) |  | 1.00 | 1.02  (0.71-1.46) | 0.87  (0.61-1.25) | 0.409 | 0.98  (0.86-1.11) | |
| Prostate(men)/Breast cancer(women) |  |  |  |  |  |  |  |  |  |  |  | |
| No.of cases/total subjects | 93/10802 | 136/12864 | 168/11993 |  |  |  | 211/19442 | 239/23835 | 215/23835 |  |  | |
| Crude HR (95%CI) | 1.00 | 0.88  (0.68-1.14) | 0.89  (0.69-1.14) | 0.388 | 0.95  (0.86-1.05) |  | 1.00 | 0.95  (0.78-1.14) | 0.87  (0.71-1.06) | 0.163 | 0.94  (0.88-1.01) | |
| Multivariable adjusted HR (95%CI) | 1.00 | 0.88  (0.68-1.15) | 0.87  (0.67-1.12) | 0.301 | 0.94  (0.85-1.04) |  | 1.00 | 0.96  (0.79-1.16) | 0.88  (0.72-1.07) | 0.209 | 0.95  (0.88-1.02) | |

^1^Adjusted for education level (less than high school, high school, college or above and missing), smoking status (nonsmoker, ex-smoker, and current smoker, missing), total energy intake (tertiles), and family history of cancer (yes, no, missing).

^2^ The test for trend was calculated with the median score for each category of cancer prevention guideline as a continuous variable.
