# Supplementary material for: Adherence to the World Cancer Research Fund/American Institute for Cancer Research and Korean Cancer Prevention Guidelines and cancer risk: a prospective cohort study from the Health Examinees-Gem study
Source: Epidemiol Health. 2023 Aug 1;45:e2023070. doi: 10.4178/epih.e2023070 (PMC10667577; doi:10.4178/epih.e2023070)
Supplement: Supplement Material 7. — Hazard ratios (HRs) and 95% confidence intervals (CI) for sensitivity analysis of cancer risk according to Korean cancer prevention guideline adherence score categories1 [file epih-45-e2023070-Supplementary-7.docx]

Supplementary Material 7. Hazard ratios (HRs) and 95% confidence intervals (CI) for sensitivity analysis of cancer risk according to Korean cancer prevention guideline adherence score categories^1^

|  | Men (n=35,659) | | | | |  | Women (n=67,112) | | | | |  |
| --- | --- | --- | --- | --- | --- | --- | --- | --- | --- | --- | --- | --- |
|  | Tertile1 | Tertile2 | Tertile3 | *p* for trend^2^ | Continuous  (per 1-point increase in score) |  | Tertile1 | Tertile2 | Tertile3 | *p* for trend^2^ | Continuous  (per 1-point increase in score) | |
| Score range | 0≤score<2.50 | 2.50≤score<3.25 | score≥3.25 |  |  |  | 0≤score<3.50 | 3.50≤score<4.00 | score≥4.00 |  |  | |
| Person year | 100815.10 | 106004.30 | 111930.90 |  |  |  | 194048.70 | 151636.30 | 256080.80 |  |  | |
| Total cancer |  |  |  |  |  |  |  |  |  |  |  | |
| No.of cases/total subjects | 688/11232 | 787/11912 | 776/12515 |  |  |  | 1082/21638 | 818/16843 | 1371/28631 |  |  | |
| Crude HR (95%CI) | 1.00 | 0.94  (0.85-1.05) | 0.78  (0.7-0.87) | <.001 | 0.89  (0.85-0.93) |  | 1.00 | 0.97  (0.89-1.06) | 0.96  (0.89-1.04) | 0.323 | 0.97  (0.93-1.02) | |
| Multivariable adjusted HR (95%CI) | 1.00 | 0.94  (0.85-1.04) | 0.78  (0.7-0.87) | <.001 | 0.89  (0.85-0.93) |  | 1.00 | 0.97  (0.88-1.06) | 0.95  (0.88-1.03) | 0.212 | 0.97  (0.92-1.01) | |
| Stomach cancer |  |  |  |  |  |  |  |  |  |  |  | |
| No.of cases/total subjects | 134/11232 | 135/11912 | 124/12515 |  |  |  | 100/21638 | 66/16843 | 161/28631 |  |  | |
| Crude HR (95%CI) | 1.00 | 0.85  (0.67-1.08) | 0.67  (0.52-0.86) | 0.001 | 0.82  (0.73-0.93) |  | 1.00 | 0.86  (0.63-1.17) | 1.23  (0.96-1.57) | 0.054 | 1.14  (0.99-1.32) | |
| Multivariable adjusted HR (95%CI) | 1.00 | 0.84  (0.66-1.07) | 0.65  (0.51-0.84) | 0.001 | 0.82  (0.73-0.92) |  | 1.00 | 0.86  (0.63-1.18) | 1.24  (0.96-1.59) | 0.048 | 1.15  (0.99-1.32) | |
| Colorectal cancer |  |  |  |  |  |  |  |  |  |  |  | |
| No.of cases/total subjects | 100/11232 | 94/11912 | 95/12515 |  |  |  | 105/21638 | 80/16843 | 107/28631 |  |  | |
| Crude HR (95%CI) | 1.00 | 0.80  (0.60-1.07) | 0.70  (0.53-0.93) | 0.019 | 0.90  (0.79-1.02) |  | 1.00 | 0.99  (0.74-1.32) | 0.77  (0.58-1.00) | 0.037 | 0.83  (0.72-0.97) | |
| Multivariable adjusted HR (95%CI) | 1.00 | 0.80  (0.60-1.07) | 0.70  (0.52-0.93) | 0.017 | 0.89  (0.78-1.02) |  | 1.00 | 0.98  (0.73-1.31) | 0.75  (0.57-0.98) | 0.023 | 0.82  (0.70-0.95) | |
| Lung cancer |  |  |  |  |  |  |  |  |  |  |  | |
| No.of cases/total subjects | 96/11232 | 91/11912 | 55/12515 |  |  |  | 74/21638 | 44/16843 | 90/28631 |  |  | |
| Crude HR (95%CI) | 1.00 | 0.72  (0.54-0.97) | 0.35  (0.25-0.48) | <.001 | 0.66  (0.57-0.75) |  | 1.00 | 0.79  (0.54-1.15) | 0.94  (0.69-1.27) | 0.820 | 1.04  (0.87-1.25) | |
| Multivariable adjusted HR (95%CI) | 1.00 | 0.74  (0.56-0.98) | 0.36  (0.26-0.51) | <.001 | 0.67  (0.58-0.77) |  | 1.00 | 0.77  (0.53-1.12) | 0.89  (0.65-1.22) | 0.617 | 1.01  (0.84-1.22) | |
| Prostate(men)/Breast cancer(women) |  |  |  |  |  |  |  |  |  |  |  | |
| No.of cases/total subjects | 88/11232 | 133/11912 | 176/12515 |  |  |  | 217/21638 | 173/16843 | 275/28631 |  |  | |
| Crude HR (95%CI) | 1.00 | 1.13  (0.86-1.48) | 1.17  (0.90-1.51) | 0.267 | 1.07  (0.96-1.20) |  | 1.00 | 1.02  (0.83-1.24) | 0.96  (0.81-1.15) | 0.638 | 0.98  (0.89-1.08) | |
| Multivariable adjusted HR (95%CI) | 1.00 | 1.11  (0.85-1.45) | 1.12  (0.87-1.45) | 0.433 | 1.06  (0.94-1.18) |  | 1.00 | 0.98  (0.80-1.20) | 0.91  (0.76-1.09) | 0.297 | 0.95  (0.86-1.05) | |

^1^Adjusted for education level (less than high school, high school, college or above and missing), smoking status (nonsmoker, ex-smoker, and current smoker, missing), total energy intake (tertiles), and family history of cancer (yes, no, missing).

^2^ The test for trend was calculated with the median score for each category of cancer prevention guideline as a continuous variable.
